# Conditional DnaB Protein Splicing Is Reversibly Inhibited by Zinc in Mycobacteria

**DOI:** 10.1128/mBio.01403-20

**Published:** 2020-07-14

**Authors:** Daniel Woods, Sweta Vangaveti, Ikechukwu Egbanum, Allison M. Sweeney, Zhong Li, Valjean Bacot-Davis, Danielle S. LeSassier, Matthew Stanger, Gabrielle E. Hardison, Hongmin Li, Marlene Belfort, Christopher W. Lennon

**Affiliations:** a Department of Biological Sciences, University at Albany, Albany, New York, USA; b The RNA Institute, University at Albany, Albany, New York, USA; c Department of Biology, Murray State University, Murray, Kentucky, USA; d Wadsworth Center, New York State Department of Health, Albany, New York, USA; e Renaissance School of Medicine, Stony Brook University, Stony Brook, New York, USA; f Signature Science, LLC, Austin, Texas, USA; University of Minnesota Medical School

**Keywords:** conditional protein splicing, DNA helicase, intein, mycobacteria

## Abstract

Inteins, as posttranslational regulatory elements, can tune protein function to environmental changes by conditional protein splicing (CPS). Translated as subdomains interrupting host proteins, inteins splice to scarlessly join flanking sequences (exteins). We used DnaB-intein1 (DnaBi1) from a replicative helicase of Mycobacterium smegmatis to build a kanamycin intein splicing reporter (KISR) that links splicing of DnaBi1 to kanamycin resistance. Using expression in heterologous Escherichia coli, we observed phenotypic classes of various levels of splicing-dependent resistance (SDR) and related these to the insertion position of DnaBi1 within the kanamycin resistance protein (KanR). The KanR-DnaBi1 construct demonstrating the most stringent SDR was used to probe for CPS of DnaB in the native host environment, M. smegmatis. We show here that zinc, important during mycobacterial pathogenesis, inhibits DnaB splicing in M. smegmatis. Using an *in vitro* reporter system, we demonstrated that zinc potently and reversibly inhibited DnaBi1 splicing, as well as splicing of a comparable intein from Mycobacterium leprae. Finally, in a 1.95 Å crystal structure, we show that zinc inhibits splicing through binding to the very cysteine that initiates the splicing reaction. Together, our results provide compelling support for a model whereby mycobacterial DnaB protein splicing, and thus DNA replication, is responsive to environmental zinc.

## Abstract (precis)

Inteins are present in a large fraction of prokaryotes and localize within conserved proteins, including the mycobacterial replicative helicase DnaB. In addition to their extensive protein engineering applications, inteins have emerged as environmentally responsive posttranslational regulators of the genes that encode them. While several studies have shown compelling evidence of conditional protein splicing (CPS), examination of splicing in the native host of the intein has proven to be challenging. Here, we demonstrated through a number of measures, including the use of a splicing-dependent sensor capable of monitoring intein activity in the native host, that zinc is a potent and reversible inhibitor of mycobacterial DnaB splicing. This work also expands our knowledge of site selection for intein insertion within nonnative proteins, demonstrating that splicing-dependent host protein activation correlates with proximity to the active site. Additionally, we surmise that splicing regulation by zinc has mycobacteriocidal and CPS application potential.

## INTRODUCTION

Inteins are intervening polypeptide domains that conditionally catalyze their own excision from flanking exteins, rendering a functional host protein. They are found in all domains of life, are particularly abundant in prokaryotes, and tend to cluster in the active sites (ASs) of essential proteins involved in DNA transactions (1, 2). Although long considered nothing more than the products of parasitic genetic elements (3), recent work has shown that some inteins function as environmental sensors to modulate host protein function in response to environmental changes (4–15). Identifying cellular or environmental factors that influence intein splicing, and thus regulation of essential protein functions, is an emerging area of interest in both basic and applied research fields (16–18).

For instance, inteins can serve as potential antimicrobial targets, as they interrupt essential proteins of numerous pathogenic bacteria and fungi but are absent from metazoan eukaryotes (19–21). One such example is represented by mycobacteria, where inteins are abundant (22). The global burden of disease caused by pathogens such as Mycobacterium tuberculosis and Mycobacterium leprae is immense. Moreover, ∼170 species of mycobacteria that do not cause tuberculosis or leprosy, termed nontuberculosis mycobacteria, amplify global and regional mycobacterial disease burdens but remain understudied (23, 24).

Mycobacterium smegmatis, a nonpathogenic model for studying mycobacteria, carries two inteins in DnaB (DnaBi1 and DnaBi2). DnaB, a hexameric helicase that denatures double-stranded DNA by ATP hydrolysis, is essential for DNA replication and thus for survival (25). Additionally, DNA helicases (DnaB in bacteria and MCM in archaea) are the most common intein-hosting proteins (26). In M. smegmatis, DnaBi1 lacks a homing endonuclease (HEN) domain and is located in the ATP-binding P-loop, and its sequence and insertion site are evolutionarily conserved in some mycobacteria, including M. leprae (7). DnaBi2 contains a HEN domain for horizontal transfer, is located within the H4 motif/DNA-binding loop of DnaB, and has sequence and insertion site conservation in M. tuberculosis (7). Given their critical locations within DnaB of M. smegmatis, both inteins likely must splice for functional helicase activity.

Inteins from the DNA replication machinery were key to identifying the mechanistic steps corresponding to the three known classes of intein splicing (27–32). Conserved nucleophilic amino acids (AAs) within the structural context of the intein and residues of adjacent exteins are central to the chemistry of breaking and making peptide bonds during splicing. DnaBi1 utilizes a noncanonical mechanism known as class 3 splicing where an internal cysteine (C118), rather than a cysteine or serine at the first position of the intein, performs the initial nucleophilic attack (33). The formation of two sequential branched intermediates during DnaBi1 splicing requires, among other features, a serine as the first C-extein residue (+1S) (33). Mutation of C118 blocks DnaBi1 excision, leading to accumulation of the unspliced and nonfunctional DnaB precursor (7). Protein splicing of M. smegmatis DnaBi1 was recently shown to be responsive to oxidative stress, whereby a reversible intramolecular disulfide bond forms between C118 and another cysteine within the intein (C48) (7). To date, that study is the only one to have reported examination of splicing regulation in the native host.

In this work, we describe the construction of an *in vivo* splicing reporter (SR) and demonstrate the cost of DnaBi1 invasion of the kanamycin resistance (KanR) protein while identifying a construct that strictly confers survival in a splicing-dependent manner. Many inteins are evolutionarily maintained in the active site of their natural host proteins (1), and interestingly, kanamycin resistance is dependent on splicing when the DnaBi1 is inserted near the KanR active site. Although no additional enzyme or cofactors are known to be required for splicing, metal ions have been found to inhibit splicing both *in vivo* and *in vitro* and to be bound to the active site of intein crystals (21, 34, 35). Using this reporter, we sought to examine whether zinc, a biologically relevant cation long known to inhibit protein splicing (34), blocks DnaBi1 splicing both *in vivo* and *in vitro*. We demonstrated that zinc reduces the survival of M. smegmatis in the presence of kanamycin when expressing a splicing-dependent DnaBi1-KanR sensor. Using an *in vitro* splicing reporter, we showed that zinc is a potent and reversible inhibitor of M. smegmatis DnaBi1 splicing and also inhibits splicing of an equivalent M. leprae DnaB intein. Finally, we present the crystal structure of zinc-bound DnaBi1, illustrating metal ion coordination mediated by the conserved initiating nucleophile of the intein required for catalysis. This work suggests that some mycobacteria regulate protein splicing in response to excess zinc, reversibly pausing DNA replication under stress.

## RESULTS

### A splicing-dependent selectable marker for use in mycobacteria.

**FIG 1:**
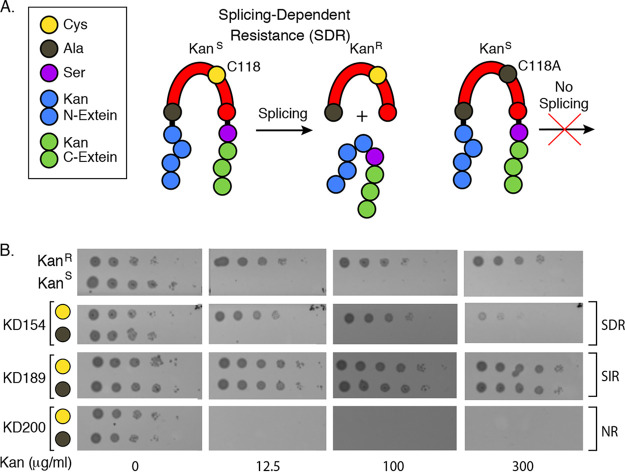
Kanamycin-DnaBi1 (KD) constructs sort into three phenotypic classes. (A) KD schematic demonstrating splicing-dependent resistance (SDR). N-exteins (blue) and C-exteins (green) were interrupted with DnaBi1 (red) from M. smegmatis at native serine residues (+1S [purple]) of KanR protein. The WT nucleophilic cysteine at position 118 (C118 [yellow]) attacks the amino terminal alanine residue (gray) to initiate splicing. Mutation of C118 to alanine renders a splicing-inactive mutant. (B) Three phenotypes emerge from KD constructs. M. smegmatis DnaBi1 was cloned into the KanR protein at one residue before each of 16 native serine residues of KanR protein that serve as +1S. A nonsplicing C118A variant of each KD construct served as a control for splicing. Growth of E. coli-containing KD constructs in the presence of increasing kanamycin concentrations resulted in splicing-dependent or splicing-independent resistance (SDR [KD154] or SIR [KD189], respectively) and in no-resistance (NR [KD200]) phenotypes. Representative titers corresponding to kanamycin concentrations (0, 12.5, 100, and 300 μg/μl) are shown here. E. coli cells expressing uninterrupted KanR and empty vector (KanS) served as positive and negative controls for kanamycin resistance, respectively.

We sought to build a splicing-dependent reporter by interrupting the bacterial aminoglycoside phosphotransferase gene (encoding the KanR protein) with the DnaBi1 intein from M. smegmatis, such that resistance to kanamycin was dependent on DnaBi1 splicing (Fig. 1A). Termed KISR (kanamycin intein splicing reporter), this reporter can be used as a tool to search for potential factors that affect intein splicing. First, we chose M. smegmatis DnaBi1 as the intein to be used to construct our KanR-DnaBi1 (KD) fusions because inteins are abundant both in mycobacteria and in DNA helicases. Second, DnaBi1 splices slowly *in vitro*, allowing us to examine the precursor protein before splicing, and is subject to conditional protein splicing (CPS) in the presence of hydrogen peroxide (7). Third, DnaBi1 is present in the human pathogen M. leprae, and the +1 residue (first amino acid of the native C-terminal DnaB extein) for both M. tuberculosis and M. leprae is serine. Finally, although intein splicing has been studied in depth *in vitro* and in nonnative cellular systems, KISR provides an avenue to explore cellular or environmental factors that influence protein splicing in the native host environment.

KanR-intein fusions have been previously developed for the purpose of directed evolution of inteins (36–38). In one such KanR-intein fusion, serine 189 (Ser189) of KanR was utilized as the +1 nucleophile for a DnaE *trans*-splicing split intein system (36). In this construct, the initiating nucleophile of DnaE was required for resistance to kanamycin. We therefore chose to insert DnaBi1 between residues 188 and 189 of KanR, with Ser189 as the predicted +1 nucleophile. We refer to this fusion as KD189 (KanR-DnaBi1-Ser189 + 1) and to its splicing-inactive variant, where the internal nucleophilic cysteine required for splicing is mutated to alanine, as KD189^C118A^. Following construction of the KD189 and KD189^C118A^ fusions, we measured resistance to kanamycin using quantitative spot titer assays in Escherichia coli. Ten-fold dilutions of cells at an optical density at 600 nm (OD_600_) of 1.0 expressing KanR, KD189, or KD189^C118A^ or a kanamycin-sensitive (KanS) negative control were spotted onto plates with increasing concentrations of kanamycin. Unexpectedly, both KD189 and splicing-inactive KD189^C118A^ provided high levels of resistance to kanamycin (Fig. 1B), in contrast to the previously developed *trans*-splicing KanR fusion (36).

We therefore inserted DnaBi1 (wild-type [WT] and C118A variants) at each of the other 15 serine residues within KanR. Importantly, all strains were able to tolerate the intein in the absence of antibiotic, and three phenotypic classes emerged from the suite of KD constructs (Fig. 1B): (i) splicing-dependent resistance (SDR), (ii) splicing-independent resistance (SIR), and (iii) no resistance (NR; kanamycin sensitive). Kanamycin resistance for the SDR class is strictly dependent on the catalytic activity of DnaBi1, represented here by KD154, and kanamycin sensitivity was found to be associated with the presence of the C118A mutation (Fig. 1B). The members of the SIR class of KD constructs resist kanamycin, whereas the members of the NR class are sensitive, with and without the C118A mutation, represented here by KD189 and KD200, respectively (Fig. 1B).

### DnaBi1 insertion position affects graded kanamycin resistance across KD classes.

**FIG 2:**
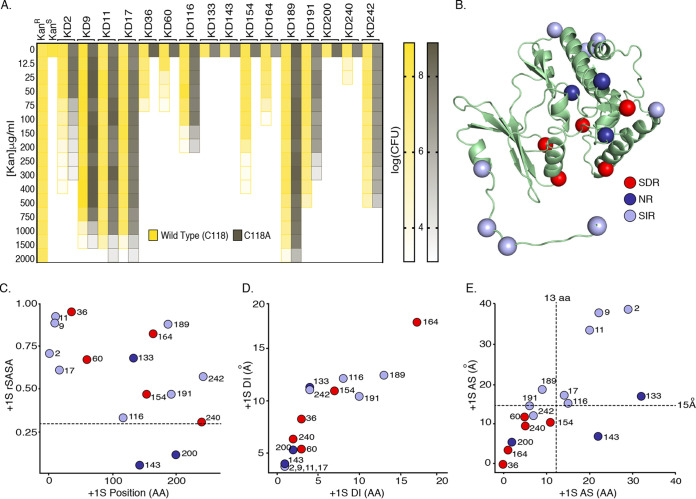
DnaBi1 insertion site selection and characteristics. (A) Graded resistance to increasing levels of kanamycin is affected by the position of insertion of DnaBi1. Data represent quantitative spot titers of CFU (right) for each of 16 KD constructs (top) with splicing-active (yellow) and splicing-inactive (gray) mutants challenged with increasing kanamycin concentrations (left; 12.5 to 2,000 μg/ml). CFU data are graphed adjacent to the uninterrupted positive control (KanR) and the empty vector negative control (KanS). Values are based on results from three biological replicates. Raw CFU data for each construct (WT and C118A) at each kanamycin concentration can be found in Table S3. (B) Serine residues for DnaBi1 insertion. The positions of the +1S residues of the SDR (red), NR (dark blue), and SIR (gray-blue) phenotypes are highlighted in the structure of KanR protein (PDB ID: 4FEW). (C) Fourteen of 16 (87.5%) of KanR +1S are more than 30% surface exposed. The relative solvent-accessible surface area (rSASA) of each serine residue (+1S) used for DnaBi1 insertion as a function of its position within the primary amino acid sequence of the KanR protein is illustrated. The exposure threshold of 30% is indicated with a horizontal dashed line. (D) Relative distances from +1S to dimer interface (DI) of KanR. The distance of each +1S residue from the DI of KanR (measured in angstroms [Å]) is plotted against the distance of each +1S from the DI along the amino acid (AA) sequence of KanR. (E) Proximity of +1S to KanR active site (AS). The distance from each +1S residue to the KanR AS (measured in angstroms [Å]) is plotted against the distance of each +1S from the KanR AS along the amino acid (AA) sequence of KanR.

Using quantitative spot titers, we examined the effects on survival and on kanamycin resistance in all 16 KD fusions (and respective splicing-inactive variants) by challenging them with increasing kanamycin concentrations (Fig. 2A). Generally, the members of the SIR class of KD constructs (KD2, KD9, KD11, KD17, KD116, KD189, KD191, and KD242) resisted high (∼1.0 mg/ml) kanamycin concentrations, albeit at levels less than those of the uninterrupted KanR control (>2.0 mg/ml). The members of the NR class (KD133, KD143, and KD200) were sensitive to the lowest kanamycin concentration tested (12.5 μg/ml), with or without splicing. The KD SDR constructs (KD36, KD60, KD154, KD164, and KD240) required splicing to confer resistance to kanamycin and survived moderately variable concentrations of kanamycin (50 to 250 μg/ml) only upon DnaBi1 splicing.

The only inference we were able to make is that, for most of the SIR mutants, DnaBi1 was inserted near the amino termini (KD2, KD9, KD11, and KD17) or carboxy termini (KD189, KD191, and KD242) of KanR, such that the intein was tolerated independently of splicing and that resistance persists at high kanamycin concentrations (Fig. 2A and B). In contrast, most of the members of the NR and SDR classes of KD constructs clustered around the middle of KanR. We therefore sought to more rigorously analyze the phenotypic classes relative to the structure of KanR.

### Splicing-dependent resistant KanR-DnaBi1 fusions cluster near the active site.

Successful construction of an intein-based, splicing-dependent reporter using a nonnative host protein requires retention of splicing capacity from within the folds of unrelated flanking sequences and may be correlated with structural parameters such as the active site and/or proximity to multimerization or the interaction partner interface (38). Mapping the position of all 16 KanR serine residues (Fig. 2B; PDB identifier [ID]: 4FEW), we calculated the relative solvent-accessible surface area (rSASA) of each +1S, the distance from the dimer interface (DI), and the distance from the active site in the context of the KanR structure. The rSASA of each +1S residue was high (>0.3 for 14 of 16 or 87.5% of the cases), indicating that the majority of the +1S residues are surface exposed (Fig. 2C) and that solvent accessibility alone shows a poor correlation with observed phenotypes. Likewise, the distance of the +1S residues from the DI, based on amino acid (AA) sequence and tertiary structure (Å), is a weak metric for correlating the observed phenotypes (SDR, SIR, and NR) of the KD constructs (Fig. 2D). Strikingly, examining the distance of the DnaBi1 insertion sites from the KanR active site (AS), based on amino acid sequence (AA) and tertiary structure (A), we found that for residues within 14 amino acids and within 16 Å (Fig. 2E), the KD constructs invariably belonged to the SDR phenotypic class. In contrast, 6 of 8 SIR mutants and 2 of 3 NR mutants were found to be inserted more remotely.

### Zinc inhibits DnaBi1 splicing in the native mycobacterial host.

**FIG 3:**
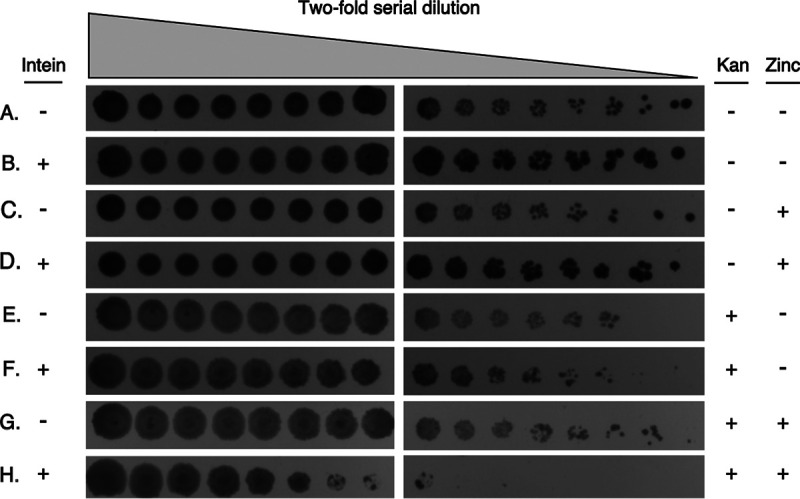
DnaBi1 splicing is inhibited by zinc in the native M. smegmatis. Two-fold dilutions of cells, starting at OD_600_ of 3 to 5, were spotted onto media in the presence of kanamycin and/or zinc as indicated. Survival of M. smegmatis expressing KD154 was selectively reduced >100-fold only in the presence of kanamycin and zinc compared to M. smegmatis expressing uninterrupted KanR. The concentration of kanamycin was 300 μg/ml and that of zinc was 100 μM where present. Three biological replicates were performed with an average reduction in survival of 170-fold (± standard deviation of 74-fold), for KD154 in the presence of 300 μg/ml kanamycin and 100 μM zinc compared to KanR lacking the intein.

Divalent cations have been shown to influence protein splicing, in some cases binding directly to the intein (21, 34, 35). Further, it has been shown that DnaBi1 is subject to conditional splicing regulation via the redox state of catalytic cysteines (7). Having built the SDR construct KD154, we hypothesized that other conditions, such as the presence of zinc (Zn^2+^; referred to here as zinc), might influence DnaB splicing. We measured the survival of M. smegmatis harboring a plasmid expressing either KanR (no intein) or KD154 (KanR + intein) in the absence of kanamycin and zinc (Fig. 3A and B), in the presence of zinc only (Fig. 3C and D), in the presence of kanamycin only (Fig. 3E and F), and, finally, in the presence of kanamycin and zinc (Fig. 3G and H). To specifically assess any role of zinc in splicing inhibition, we were careful to use concentrations of kanamycin and zinc that did not lead to a difference in survival between uninterrupted KanR (Fig. 3C and E) and the KanR-DnaBi1 fusion at position 154, KD154 (Fig. 3D and F). This measure was taken to control for any toxicity from zinc unrelated to splicing inhibition. Further, while the concentration of kanamycin used did not lead to differences in rates of survival between the cells expressing KanR and those expressing KD154, the concentration was chosen to be sufficiently high to ensure that any reduction in active enzyme levels that was due to splicing inhibition would be detectable. When both kanamycin and zinc were present in the plates, survival of M. smegmatis expressing KD154 was reduced by >100-fold, whereas survival was unaffected for the strain expressing uninterrupted KanR (Fig. 3G and H). This degree of splicing inhibition is comparable to that reported previously for H_2_O_2_ (7) and indirectly links DnaB helicase function with zinc homeostasis and redox metabolism.

### Zinc inhibits DnaBi1 splicing *in vitro*.

**FIG 4:**
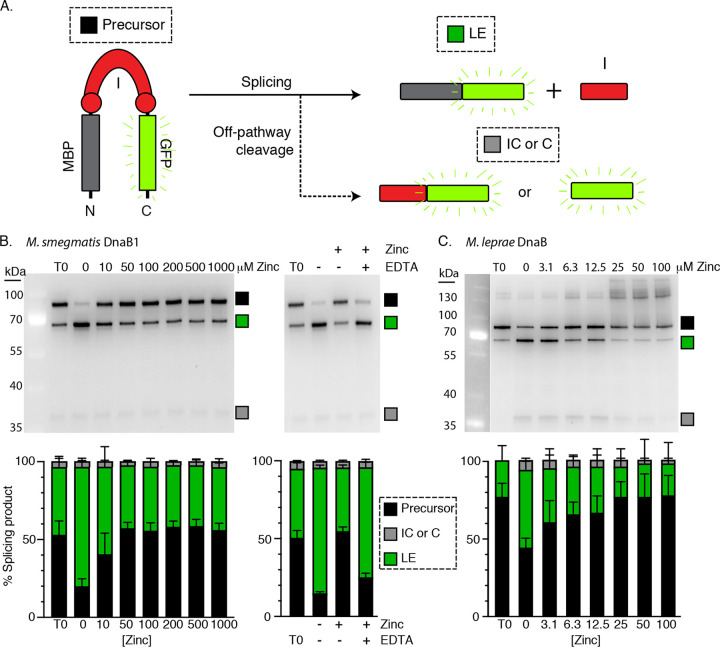
Zinc potently inhibits DnaBi1 from M. smegmatis and M. leprae *in vitro*. (A) MIG schematic. The in-gel fluorescent reporter construct with maltose-binding protein, MBP-intein-GFP (MIG), was used to monitor M. smegmatis and M. leprae DnaBi1 splicing. Fluorescent product sizes indicate precursor (P), ligated extein (LE), or off-pathway N- and C-terminal cleavage reactions. (B) Zinc reversibly inhibits M. smegmatis DnaBi1 splicing. The gel of MIG splicing shows an accumulation of precursor and a concomitant reduction of ligated exteins in the presence of zinc compared to untreated MIG (left). This inhibition was reversed in the presence of the zinc chelator EDTA (right). Results of quantitation of MIG reporter under conditions of increasing zinc levels in the presence or absence of EDTA are shown below the representative gel (stack plots) where the ratio of splice products is plotted. (C) Splicing of M. leprae intein is also inhibited by zinc. Similarly to the M. smegmatis results, zinc-treated MIG DnaBi1 from M. leprae showed an accumulation of precursor and a reduction in the levels of ligated exteins compared to the control, in the low micromolar range (top left). Data are representative of results from three biological replicates, and where error bars are present, values are expressed as averages ± standard deviations.

To demonstrate that the SDR phenotype seen under *in vivo* conditions corresponded to splicing, we tested zinc-mediated DnaBi1 splicing inhibition *in vitro*. For this, we employed a splicing reporter, MIG (maltose binding protein [MBP]-intein-green fluorescent protein [GFP]), where an intein is flanked by the nonnative exteins maltose binding protein and green fluorescence protein (DnaBi1-MIG). GFP-containing species that include a precursor, ligated exteins (LE), and C-terminal cleavage products (IC/C) can be separated by size and detected using in-gel fluorescence following seminative PAGE (10, 11) (Fig. 4A).

After DnaBi1-MIG overexpression, cell lysates were incubated with a range of zinc concentrations and splicing was monitored following incubation at 16°C for ∼16 h. Whereas splicing proceeds efficiently in the absence of zinc, DnaBi1 splicing is blocked when zinc is present, even at low micromolar concentrations (Fig. 4B, left). Intein catalysis is almost completely inhibited at 10 μM (Fig. 4B, left), suggesting that the concentration of zinc present for our *in vivo* studies (Fig. 3; 100 μM) is sufficient to block DnaBi1 splicing. We tested the reversibility of this inhibition by adding the chelator EDTA, which is a strong zinc binding agent. For these experiments, we first incubated DnaBi1-MIG with zinc for 1 h to allow binding, followed by the addition of EDTA. EDTA reversed the zinc inhibition, allowing splicing to proceed at an extent equivalent to that seen in its absence (Fig. 4B, right). This indicates that zinc binding to DnaBi1 is responsible for inhibition and that this binding, and thus inhibition, is reversible.

We next addressed whether the DnaB intein from the human pathogen M. leprae, which shares insertion site and sequence homology with M. smegmatis DnaBi1 (7), was also responsive to zinc. We observed a similar pattern of inhibition, although zinc appears to be an even more potent inhibitor for M. leprae DnaB intein splicing inhibition (Fig. 4C). However, upon incubation with zinc, we observed an increased level of off-pathway cleavage products as well as the accumulation of larger products indicative of aggregation for M. leprae DnaBi1.

### Crystal structure of DnaBi1 bound to zinc.

**FIG 5:**
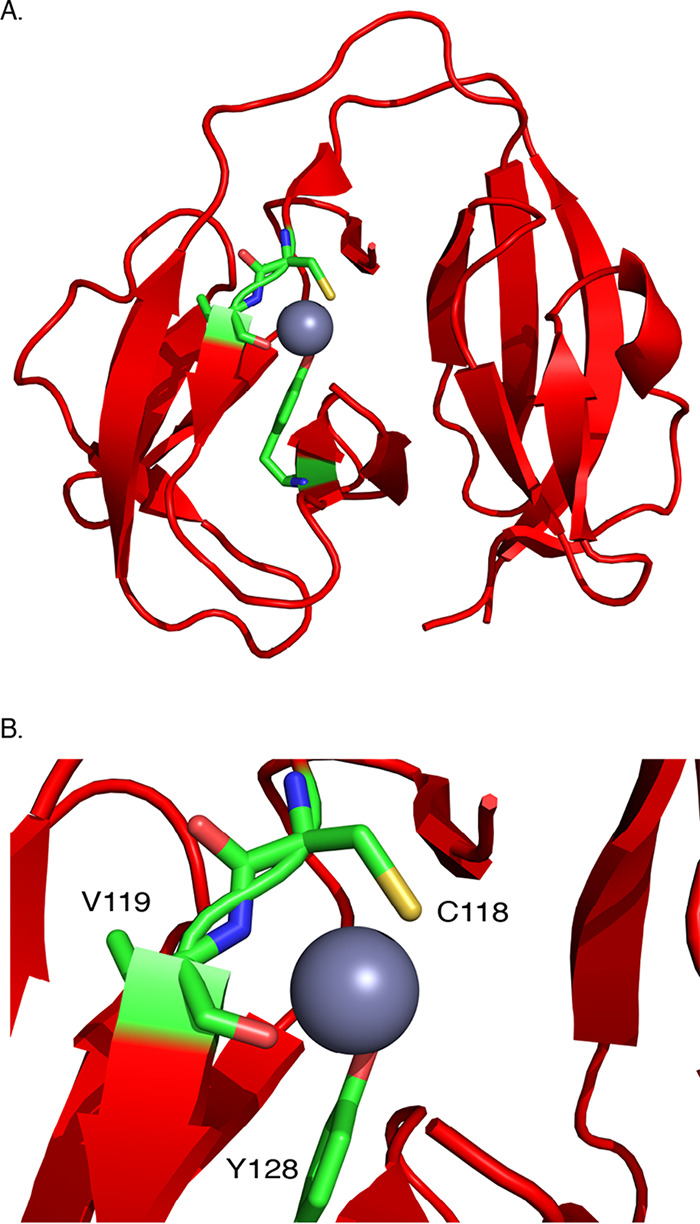
Zinc binds the catalytic center of DnaBi1. (A) Crystal structure of Mycobacterium smegmatis DnaBi1 (red) bound to zinc (gray) at 1.95 Å (PDB ID: 6OWN). A single zinc ion is coordinated by C118, V119, and Y128 (residues shown as sticks). (B) A magnification of the zinc ion and coordinating residues. C118, V119, and Y128 are colored by atom as carbon (green), nitrogen (blue), oxygen (red), and sulfur (yellow).

Recently, we reported the crystal structure of the first class 3 intein, M. smegmatis DnaBi1, which adopts a fold similar to that seen with other previously described class 1 inteins (7). We therefore set out to solve the structure of DnaBi1 bound to zinc to understand the precise nature of DnaBi1 splicing inhibition (Fig. 5A). The DnaBi1-zinc complex crystallized in monoclinic space group P1 21 1 (Table 1). The asymmetric unit consisted of five zinc ions and three DnaBi1 molecules (referred to as A, B, and C). The zinc-bound structure of DnaBi1 is nearly identical to that of the *apo* DnaBi1 (root mean square [RMS] deviation of 0.065). Among the five zinc ions, one is bound at the molecular interface between molecule A and molecule B within the three DnaBi1 molecules in the asymmetric unit. It is coordinated by residues Y49 and R110 of molecule A and by residue W14 of molecule B. The second zinc is bound in a similar fashion, although it is coordinated by residues from molecule A and a B molecule generated through crystallographic symmetry. Three other zinc ions bind to each DnaBi1 molecule in identical positions. These three zinc ions are coordinated by sulfur on C118, peptide backbone atoms (N and O) of V119, and the hydroxyl group (OH) of Y128 of each DnaBi1 (Fig. 5B). This coordination explains why zinc blocks catalysis, as the C118 is a catalytic nucleophile necessary for splicing. Additionally, V119 and Y128 are found within a conserved splicing block of DnaBi1. Consistent with our SDR results obtained using our KD154 sensor in M. smegmatis (Fig. 3), as well as our findings using the MIG reporter (Fig. 4), zinc inhibition of DnaBi1 splicing was found to occur through direct binding to the initiating nucleophile for protein splicing (Fig. 5).

**TABLE 1:** Data collection and refinement statistics

Property	Value(s)
Data collection statistics	
Space group	P 1 21 1
Cell dimensions	
*a*, *b*, *c* (Å)	64.05, 56.91, 64.73
α, β, γ (°)	90.00, 106.60, 90.00
Resolution (Å)	38.48 to 1.95
*R*_sym_	0.098 (0.762)
*I*/*σI*	9.4 (1.95)
Completeness (%)	97.0 (97.0)
Redundancy	2.6 (2.4)

Refinement statistics	
Resolution (Å)	38.48 to 1.95
No. of reflections	31,743
*R*_work_/*R*_free_	0.209/0.244
No. of molecules	3,121
Protein	2,855
Ligand/ion	5
Water	261
*B*-factors (Å^2^)	32.8
Protein	32.2
Zinc	62.6
Water	38.4

RMS deviations	
Bond lengths (Å)	0.005
Bond angles (°)	0.77

Ramachandran plot	
Favored (%)	97.3
Allowed (%)	2.7
Disallowed (%)	0

## DISCUSSION

Inteins are often found in experimentally challenging organisms such as pathogenic bacteria (e.g., M. tuberculosis) (11, 25), pathogenic fungi (e.g., Cryptococcus neoformans) (21, 35), and extremophilic archaea (e.g., Pyrococcus horikoshii) (9, 10). As a result, studies examining conditional protein splicing (CPS) in the native host have proven difficult. However, as CPS increasingly emerges as a potentially widespread mechanism of posttranslational regulation (4, 5, 8–15), tools to study protein splicing in the native host background are necessary to understand the role of inteins in nature. One fairly tractable intein-containing organism is M. smegmatis, which houses four inteins, with two in DnaB, one in GyrA, and one in PhoH. Whereas M. smegmatis is generally innocuous, its two DnaB inteins are conserved in the pathogens M. leprae (DnaBi1) and M. tuberculosis (DnaBi2), the causative agents of leprosy and tuberculosis, respectively. Here, we describe the construction of a genetic sensor that links survival of M. smegmatis to the catalytic activity of its own DnaB1 intein (Fig. 1 and 2). Using this sensor, we demonstrated that zinc inhibits DnaBi1 splicing directly in the native host (Fig. 3). Additionally, we found that zinc is a highly potent inhibitor of M. smegmatis and M. leprae DnaB intein splicing (Fig. 4). Finally, we solve the structure of the zinc-bound M. smegmatis DnaB1 intein complex, which illustrates the mechanism of zinc inhibition through binding to C118, the residue required to initiate the splicing reaction (Fig. 5).

### An *in vivo* intein splicing sensor.

An *in vivo* splicing sensor for mycobacteria was developed here to probe cellular or environmental conditions that influence protein splicing. It is not uncommon to interrupt the KanR protein with inteins. A KanR-intein system which inserted three residues, including a nonnative cysteine flanking the splice site, employed KanR to evolve an intein to splice in response to a small molecule (37). Another KanR-intein system designed for a *trans*-splicing intein, Nostoc punctiforme DnaE, was developed previously (36). However, when we inserted M. smegmatis DnaBi1 between the same KanR residues, kanamycin resistance was splicing independent. We suspect that the difference in splicing-dependent kanamycin-resistant phenotypes between the DnaE split intein and the DnaBi1 intein results from substantial differences in the mechanisms of protein splicing. While DnaE utilizes class 1 *trans*-splicing, DnaBi1 undergoes class 3 *cis*-splicing.

We therefore took an unbiased approach for achieving splicing-dependent resistance (SDR). Since serine is a conserved +1 residue for DnaBi1, each of 16 native KanR serine residues was used as +1S for insertion of DnaBi1 to create the suite of KD constructs. Using splicing-inactive (C118A) KD variants as a control, three phenotypic classes emerged under conditions of challenge with increasing kanamycin concentrations. First, the no-resistance (NR) class is the phenotypic class that blocks KanR from processing kanamycin with or without splicing capacity. Notably, +1S143 and +1S200 are the two most deeply buried serines and are those closest to the dimerization interface (Fig. 2C and D), and disrupting proper folding and dimerization are plausible explanations for this phenotype. Second, the splicing-independent resistance (SIR) phenotype shows resistance to high concentrations of kanamycin regardless of splicing capacity and, perhaps unsurprisingly, most of these insertions are at the N-terminal and C-terminal regions of KanR. Finally, there is the desired class of the splicing-dependent resistance (SDR) phenotype, where DnaBi1 interrupts a functional domain of the KanR protein such that its removal by splicing is required for kanamycin resistance. KD154 demonstrated the SDR phenotype up to relatively high kanamycin concentrations (∼200 μg/ml) and therefore represented the best KanR insertion for our *in vivo* splicing sensor. Interestingly, all of the KD-expressing strains demonstrated variable costs with respect to CFU compared to a control strain expressing uninterrupted KanR protein (Fig. 2A).

To better understand the phenotypic classes of resistance to kanamycin, we examined the structural context of the +1S used in the KanR protein. The feature-based analysis done here (Fig. 2B to E) is similar to that performed with 412 naturally occurring inteins and their precursor proteins (38). In that study, the secondary structure location, the degree of burial, and the distance from the dimerization site and the active site were all significantly different for native intein insertion sites compared to all cysteine/serine/threonine residues present in the precursor proteins. In this work, which was with a more limited sample size, we found that the distance from the active site is the distinctive feature that differentiates the SDR and NR residues from the SIR residues in the KD constructs and can guide site selection strategies for intein-based protein engineering.

### Zinc inhibits DnaBi1 *in vivo* and *in vitro*.

Using the KD154 reporter, DnaBi1 splicing was previously shown to be inhibited by the reactive oxygen species (ROS) hydrogen peroxide in M. smegmatis (7). Additionally, several *in vitro* measures demonstrated that DnaBi1 splicing is directly inhibited by ROS and reactive nitrogen species. Mechanistically, hydrogen peroxide induces the formation of a reversible disulfide bond between two cysteines (C48 and C118) within DnaBi1, both of which are required for splicing activity. Pathogens such as M. leprae and M. tuberculosis encounter this stress in macrophages during pathogenesis (39, 40).

Here, with the same KD154-SDR construct, we demonstrated that zinc was able to reduce the survival of M. smegmatis in response to the presence of zinc and kanamycin in an intein-dependent manner by >100-fold (Fig. 3). Importantly, the concentration of zinc or kanamycin alone did not reduce survival. This strongly suggests that zinc directly inhibits DnaBi1 splicing in M. smegmatis, the natural host of the intein.

Divalent cations, in particular, zinc, platinum, and copper, have previously been shown to inhibit protein splicing of several inteins *in vitro* (34, 35). Additionally, hedgehog autoprocessing in eukaryotes, which exhibites evolutionary, structural, and mechanistic similarities to protein splicing, is inhibited by zinc (41). Further, the platinum-based anticancer drug cisplatin has been shown to inhibit splicing of two inteins from pathogens, the RecA intein from M. tuberculosis (19) and the Prp8 intein from Cryptococcus neoformans (21). Additionally, overexpression of the M. tuberculosis RecA intein increases survival in response to cisplatin, suggesting binding within the mycobacterial cellular environment (20). Zinc represents an intriguing candidate to block splicing. From the mechanistic perspective, zinc is not redox active, and thus cysteine oxidation is not required to block splicing, in contrast to copper (35). Presumably, reversible binding to the intein could pause splicing when zinc levels are high and could then be naturally titrated away as cellular levels decrease to allow splicing to proceed. The concentration of zinc in mycobacteria is thought to range from 10 to 1,000 μM (42–44) which fits well with our model that splicing proceeds under low-zinc conditions but is reversibly blocked under conditions of high zinc concentrations (e.g., 100 μM).

Metals, including zinc, are used by macrophages as a general strategy in defense against pathogens (42, 43, 45, 46). The concentration of zinc increases following M. tuberculosis infections in both mouse and human macrophage phagolysosomes (42, 46). Additionally, M. tuberculosis expresses the zinc efflux pump *ctpC* during human macrophage infection (46). While DnaBi1 is not conserved in M. tuberculosis (7), splicing of the M. tuberculosis RecA intein is inhibited by zinc (34, 47), suggesting that M. tuberculosis protein splicing may be responsive to zinc during infection. Regardless, as DnaBi1 is conserved in M. leprae, these results provide insights into how M. leprae DnaB intein splicing may respond to host defense.

Using the MIG system, we found that zinc is a potent inhibitor of splicing from both the M. smegmatis and M. leprae DnaB inteins (Fig. 4). Our results suggest a high affinity for zinc, with strong splicing inhibition at low micromolar concentrations. We further demonstrated zinc binding by solving the crystal structure of M. smegmatis DnaBi1 bound to zinc, which clearly illustrates the mechanism of inhibition through interaction with the catalytic nucleophile C118 (Fig. 5). Together, the results from these *in vitro* studies confirm and expand upon our *in vivo* work in M. smegmatis (Fig. 3).

Inteins hold tremendous potential as posttranslational regulators, and as the possible biological roles of inteins are being investigated more carefully, intriguing examples of CPS are now frequently being discovered. The M. smegmatis DnaB intein is centrally positioned within the active site of the ATPase domain, implying that prior to splicing, DnaB function is compromised. Given the absolute requirement for functional DnaB in replication, control of splicing of this one intein could serve to regulate a critical cellular process such as replication. We hypothesize that the DnaB1 intein of M. smegmatis is capable of responding to a variety of environmental cues, including oxidative and metal stress, allowing the cell to pause protein splicing and thus replication until environmental conditions improve to permit intein activity.

## MATERIALS AND METHODS

### Bacterial strains and growth conditions.

Escherichia coli NEB5α (New England BioLabs) and BL21(DE3) (Novagen) were grown in LB broth, Miller (Difco), with aeration at 250 rpm. Plasmids were transformed into cells by electroporation, and transformants were selected by plating on LB agar with the appropriate antibiotic and were incubated at 37°C overnight. For work in M. smegmatis, MC^2^155 was used (K. Derbyshire) and the conditions employed are described below.

### Construction of plasmids.

All plasmids used in the present study are listed in Table S1 in the supplemental material, and all oligonucleotides, synthesized by Integrated DNA Technologies, are listed in Table S2. EZ-Vision DNA dye (Amresco) was used to visualize DNA. CloneAmp HiFi PCR Premix (Clontech) was used to amplify DNA. M. smegmatis DnaBi1 was inserted in-frame using Gibson assembly (NEB) into *kanR* [aminoglycoside *O*-phosphotransferase APH(3′)-Ia; NCBI reference sequence WP_000018329.1] of pUC4K. An E.Z.N.A. plasmid minikit (Omega) was used to prepare plasmids. Construction of the maltose binding protein (MBP)-intein-green fluorescent protein (GFP) (MIG) reporter for M. smegmatis and M. leprae DnaBi1 was previously described (7). Within MIG, the intein is flanked by 8 N-extein residues and 10 C-extein residues from native DnaB sequence. All clones were verified by sequencing (EtonBio).

10.1128/mBio.01403-20.1 TABLE S1 Plasmids used in this study. Download Table S1, DOCX file, 0.02 MB. Copyright © 2020 Woods et al. 2020 Woods et al. This content is distributed under the terms of the Creative Commons Attribution 4.0 International license.

10.1128/mBio.01403-20.2 TABLE S2 Oligonucleotides used in this study. Download Table S2, DOCX file, 0.02 MB. Copyright © 2020 Woods et al. 2020 Woods et al. This content is distributed under the terms of the Creative Commons Attribution 4.0 International license.

10.1128/mBio.01403-20.3 TABLE S3 Raw CFU data for KanR, KanS, and KD fusions with means of results from 3 independent experiments ± standard deviations. Download Table S3, DOCX file, 0.04 MB. Copyright © 2020 Woods et al. 2020 Woods et al. This content is distributed under the terms of the Creative Commons Attribution 4.0 International license.

### Kanamycin intein splicing reporter spot titers.

Each of 16 KanR serine residues was used as +1 nucleophile, and the nomenclature denotes KanR-DnaBi1 (KD) and position of native KanR serine (e.g., KD154). KD fusions were screened in E. coli to identify candidates that required splicing for survival on kanamycin-containing media as demonstrated by a splicing-inactive KD C118A variant. All 16 KD constructs and their splicing-inactive counterparts were grown on LB media containing carbenicillin (50 μg/ml) and various amounts of kanamycin (0, 12.5, 25, 50, 75, 100, 150, 200, 250, 300, 400, 500, 750, 1,000, 1,500, or 2,000 μg/ml) and were phenotypically defined as representing splicing-dependent resistance, splicing-independent resistance, or no resistance (SDR, SIR, or NR, respectively). The degree of survivability is reported as CFU per kanamycin concentration.

### *In silico* analysis of KanR insertion sites.

KanR belongs to the family of aminoglycoside-3′-phosphotransferase [APH(3′)]. The three-dimensional (3D) structure of KanR [APH(3′) Ia; PDB ID: 4FEW], bound to kanamycin (48), shows that KanR exists as a dimer. We used this 3D structure to analyze the context and properties of the +1 serine residues in KanR. To assess the intein insertion site characteristics, we calculated the relative solvent-accessible surface area (rSASA), the distance to the dimer interface, and the distance to the active site for each of the serine residues in KanR. The rSASA for a residue is the solvent-accessible surface area of the residue in the context of the protein (taking into account its neighboring two residues) relative to a fully exposed residue. rSASA values were calculated using PyMOL (version 2.3.2; Schrödinger, LLC) and ranges from 0.0 to 1.0, indicating a completely buried residue and a fully exposed residue, respectively. Residues with rSASA values of 0.3 or higher are considered representative of solvent exposure. The dimer interface (DI) is defined by all residues of the KanR subunits within 5 Å of each other in the dimer structure. The active site (AS) is defined by all residues of KanR within 5 Å of the bound kanamycin or ATP molecule in the structure. To understand the effects of the proximity of the +1S to the DI or AS, the number of amino acids (AA) between a given serine residue and the closest amino acid residue in the active site or the dimer interface and the shortest distance in three-dimensional space (expressed in angstroms [Å]) between a given serine residue and the residues in the active site and the dimer interface were calculated using the sequence and the structure of KanR, respectively.

### M. smegmatis spot titers.

M. smegmatis (MC^2^155) was grown in standard 7H9 (liquid) and 7H10 (solid) media prior to spot titer assays. For studies using M. smegmatis, KD154 was chosen as the best candidate for a splicing-dependent reporter due to its ability to resist relatively high concentrations of kanamycin (∼150 μg/ml) and was subsequently subcloned into a mycobacterial shuttle vector, pMBC283, using Gibson assembly (NEB) as previously described (7). For spot titer studies, 7H10 was prepared without albumin or catalase. Two-fold dilutions, starting from OD_600_ values of 3 to 5, of cells were spotted. The concentrations of cells, zinc acetate, and kanamycin used are described in the figure legends. Cells present at 1.25 μl at each titer were spotted and grown at 30°C for 5 days.

### MIG splicing assay.

Zinc-mediated inhibition of M. smegmatis DnaBi1 and M. leprae DnaB intein splicing was measured using our MIG reporter (11). MIG was expressed using BL21(DE3). For overexpression assays, overnight cultures grown in LB were subcultured 1:100 in fresh LB, grown to an OD_600_ of ∼0.5 at 37°C, moved to 30°C, and induced with 0.5 mM isopropyl β-d-1-thiogalactopyranoside (Gold Bio), and protein expression was allowed to proceed for 1 h. Cell pellets were resuspended in MIG buffer (50 mM Tris [pH 8.0], 20 mM NaCl, 10% glycerol) and lysed on ice using a tip sonicator. Samples were pelleted at 20,000 × *g*, and soluble lysate was assayed. A time zero sample was taken and stored at –20°C, while the sample lysate was incubated at 16°C for ∼16 h for M. smegmatis DnaBi1 and ∼24 h for M. leprae DnaB. Zinc acetate was added at the indicated concentrations immediately prior to incubation. To verify that zinc-mediated splicing inhibition was reversible, following 1-h incubation with zinc, samples were treated with a 2× excess of EDTA and incubated further as indicated. Samples were separated on 8% to 16% Tris-glycine gels (Bio-Rad) using Laemmli sample buffer (Bio-Rad) containing 1% β-mercaptoethanol. GFP-containing products were detected with an Amersham model 680 imager (GE Healthcare). ImageJ was used for quantitation, and GraphPad Prism (v7.02) was used for analysis.

### Crystal structure determination.

The purification and structure determination of the zinc-bound DnaBi1 were performed using methods similar to those previously used for the apo M. smegmatis DnaBi1 intein (7). Briefly, M. smegmatis DnaBi1 was purified using a chitin affinity column followed by gel filtration chromatography and was concentrated to 13 mg/ml. M. smegmatis DnaBi1-zinc crystals were grown using a hanging-drop vapor diffusion method by mixing 1 μl of DnaBi1 and 1 μl of reservoir solution containing 20% (vol/vol) 2-methyl-2,4-pentanediol, 0.1 M sodium acetate (pH 4.6), 0.2 M sodium chloride, and 2 mM zinc chloride. Crystals were grown at room temperature for approximately 1.5 weeks.

Prior to data collection, all crystals were transferred to a cryoprotectant solution containing crystallization buffer with an MPD (2-methyl-2,4-pentanediol) concentration of 30%. The crystals were flash-cooled directly in liquid nitrogen. Diffraction data for the DnaBi1-zinc crystals were collected at 100 K at the F1 beamline of the Cornell High Energy Synchrotron Source (CHESS). The images were processed in imosfilm (CCP4 version 7) and scaled using Scala (CCP4 version 7). The crystal structure of M. smegmatis DnaB1 intein-zinc was solved by molecular replacement using Phaser (Phenix version 1.12-2829) with PDB ID 6BS8 as the starting model. The structure was further refined using Phenix version 1.12-2829.

### Data collection and refinement statistics.

Data collection and refinement statistics are listed in Table 1.

### Data availability.

The final refinement of the M. smegmatis DnaBi1-zinc structure was deposited with the Worldwide Protein Data Bank under PDB ID: 6OWN.
