# Supplementary material for: Conditional DnaB Protein Splicing Is Reversibly Inhibited by Zinc in Mycobacteria
Source: mBio. 2020 Jul 14;11(4):e01403-20. doi: 10.1128/mBio.01403-20 (PMC7360933; doi:10.1128/mBio.01403-20)
Supplement: TABLE S1 [file mBio.01403-20-st001.docx]

**Supplemental Table 1** Plasmids used in this study.

| Plasmid | Features | Source |
| --- | --- | --- |
| pACYCDuet-1 | Expression vector, T7 promoter, Cam^R^ | Novagen |
| pACYC MIG SufB | Used as cloning backbone by removing M. tuberculosis SufB intein insert using ClaI/SphI | 1 |
| pACYC MIG  *M. smegmatis* DnaBi1 | *M. smegmatis* DnaB intein 1 flanked by short native extein residues (N-extein: AARPGVGK; C-extein: STLGLDFMRS) cloned into ClaI/SphI sites between MBP and GFP coding sequences in pACYCDuet-1 backbone | 2 |
| pACYC MIG *M. leprae* DnaBi1 | *M. leprae* DnaB intein 1 flanked by short native extein residues (N-extein: AARPGVGK; C-extein: SNTLGLDFMRS) cloned into ClaI/SphI sites between MBP and GFP coding sequences in pACYCDuet-1 backbone | 2 |
| pMBC238 | Mycobacterial shuttle vector | Kathleen McDonough |
| pMBC238 KanR | Mycobacterial shuttle vector positive control | 2 |
| pMBC283 KanR-DnaBi1 WT | Mycobacterial shuttle vector with KanR interrupted by *M. smegmatis* DnaBi1 wild-type at Ser154 | 2 |
| pMBC283 KanR-DnaBi1 C118A | Mycobacterial shuttle vector with KanR interrupted by *M. smegmatis* DnaBi1 C118A at Ser154 | 2 |
| puc4k | positive control for kanamycin resistance protein (KanR) | 3 |
| puc18 | negative control for kanamycin resistance protein (KanS) | 4 |
| puc4+DnaBi1 KD2 WT | splicing active DnaBi1 insertion at serine 2 of kanR protein | This study |
| puc4k +DnaBi1 KD2 C118A | inactive DnaBi1 insertion at serine 2 of kanR protein | This study |
| puc4k +DnaBi1 KD9 WT | splicing active DnaBi1 insertion at serine 9 of kanR protein | This study |
| puc4k +DnaBi1 KD9 C118A | inactive DnaBi1 insertion at serine 9 of kanR protein | This study |
| puc4k +DnaBi1 KD11 WT | splicing active DnaBi1 insertion at serine 11 of kanR protein | This study |
| puc4k +DnaBi1 KD11 C118A | inactive DnaBi1 insertion at serine 11 of kanR protein | This study |
| puc4k +DnaBi1 KD17 WT | splicing active DnaBi1 insertion at serine 17 of kanR protein | This study |
| puc4k +DnaBi1 KD17 C118A | inactive DnaBi1 insertion at serine 17 of kanR protein | This study |
| puc4k +DnaBi1 KD36 WT | splicing active DnaBi1 insertion at serine 36 of kanR protein | This study |
| puc4k +DnaBi1 KD36 C118A | inactive DnaBi1 insertion at serine 36 of kanR protein | This study |
| puc4k +DnaBi1 KD60 WT | splicing active DnaBi1 insertion at serine 60 of kanR protein | This study |
| puc4k +DnaBi1 KD60 C118A | inactive DnaBi1 insertion at serine 60 of kanR protein | This study |
| puc4k +DnaBi1 KD116 WT | splicing active DnaBi1 insertion at serine 116 of kanR protein | This study |
| puc4k +DnaBi1 KD116 C118A | inactive DnaBi1 insertion at serine 116 of kanR protein | This study |
| puc4k +DnaBi1 KD133 WT | splicing active DnaBi1 insertion at serine 133 of kanR protein | This study |
| puc4k +DnaBi1 KD133 C118A | inactive DnaBi1 insertion at serine 133 of kanR protein | This study |
| puc4k +DnaBi1 KD143 WT | splicing active DnaBi1 insertion at serine 143 of kanR protein | This study |
| puc4k +DnaBi1 KD143 C118A | inactive DnaBi1 insertion at serine 143 of kanR protein | This study |
| puc4k +DnaBi1 KD154 WT | splicing active DnaBi1 insertion at serine 154 of kanR protein | This study |
| puc4k +DnaBi1 KD154 C118A | inactive DnaBi1 insertion at serine 154 of kanR protein | This study |
| puc4k +DnaBi1 KD164 WT | splicing active DnaBi1 insertion at serine 164 of kanR protein | This study |
| puc4k +DnaBi1 KD164 C118A | inactive DnaBi1 insertion at serine 164 of kanR protein | This study |
| puc4k +DnaBi1 KD189 WT | splicing active DnaBi1 insertion at serine 189 of kanR protein | This study |

| puc4k +DnaBi1  KD189 C118A | inactive DnaBi1 insertion at serine 189 of kanR protein | This study |
| --- | --- | --- |
| puc4k +DnaBi1  KD191 WT | splicing active DnaBi1 insertion at serine 191 of kanR protein | This study |
| puc4k +DnaBi1  KD191 C118A | inactive DnaBi1 insertion at serine 191 of kanR protein | This study |
| puc4k +DnaBi1  KD200 WT | splicing active DnaBi1 insertion at serine 200 of kanR protein | This study |
| puc4k +DnaBi1  KD200 C118A | inactive DnaBi1 insertion at serine 200 of kanR protein | This study |
| puc4k +DnaBi1  KD240 WT | splicing active DnaBi1 insertion at serine 240 of kanR protein | This study |
| puc4k +DnaBi1  KD240 C118A | inactive DnaBi1 insertion at serine 240 of kanR protein | This study |
| puc4k +DnaBi1  KD242 WT | splicing active DnaBi1 insertion at serine 242 of kanR protein | This study |
| puc4k +DnaBi1  KD242 C118A | inactive DnaBi1 insertion at serine 242 of kanR protein | This study |

1. Topilina NI, Green CM, Jayachandran P, Kelley DS, Stanger MJ, Piazza CL, Nayak S, Belfort M. 2015. SufB intein of Mycobacterium tuberculosis as a sensor for oxidative and nitrosative stresses. Proc Natl Acad Sci U S A 112:10348-10353.

2. Kelley DS, Lennon CW, Li Z, Miller MR, Banavali NK, Li H, Belfort M. 2018.

Mycobacterial DnaB helicase intein as oxidative stress sensor. Nat Commun 9:4363.

3. Vieira J, Messing J. 1982. The pUC plasmids, anM13mp7-derived system for

insertion mutagenesis and sequencing with synthetic universal primers. Gene 19:259-268.

4. Norrander J, Kempe T, Messing J. 1983. Construction of improved M13 vectors using

oligonucleotide-directed mutagenesis. Gene 26:101-106.
