# Supplementary material for: Conditional DnaB Protein Splicing Is Reversibly Inhibited by Zinc in Mycobacteria
Source: mBio. 2020 Jul 14;11(4):e01403-20. doi: 10.1128/mBio.01403-20 (PMC7360933; doi:10.1128/mBio.01403-20)
Supplement: TABLE S2 [file mBio.01403-20-st002.docx]

**Supplemental Table 2** Oligonucleotides used in this study.

| Oligo ID | Sequence (5' to 3') | Application |
| --- | --- | --- |
| IDT5580 | ggttcccacgcacaacagccatattcaacgggaaacgtcttgc | pUC4K forward primer for +1S2 |
| IDT5581 | gtcgagcgcgagcgccataacaccccttgtattactgtttatgtaagcagacag | pUC4K reverse primer for +1S2 |
| IDT5582 | gtcgagcgcgagcgccataacaccccttgtattactgtttatgtaagcagacag | DnaB1 forward primer for +1S2 |
| IDT5583 | ggcaacttataccgagttgtgcgtgggaacc | DnaB1 reverse primer for +1S2 |
| IDT5584 | ggttcccacgcacaactcttgctcgaggccgcgattaaattcc | pUC4K forward primer for +1S9 |
| IDT5585 | gtcgagcgcgagcgccgtttcccgttgaatatggctcataacaccc | pUC4K reverse primer for +1S9 |
| IDT5586 | attcaacgggaaacggcgctcgcgctcgacac | DnaB1 forward primer for +1S9 |
| IDT5587 | cggcctcgagcaagagttgtgcgtgggaacc | DnaB1 reverse primer for +1S9 |
| IDT5588 | ggttcccacgcacaactcgaggccgcgattaaattccaacatgg | pUC4K forward primer for +1S11 |
| IDT5589 | gtcgagcgcgagcgcgcaagacgtttcccgttgaatatggctc | pUC4K reverse primer for +1S11 |
| IDT5590 | cgggaaacgtcttgcgcgctcgcgctcgacac | DnaB1 forward primer for +1S11 |
| IDT5591 | taatcgcggcctcgagttgtgcgtgggaacc | DnaB1 reverse primer for +1S11 |
| IDT5592 | ggttcccacgcacaactccaacatggatgctgatttatatgggtataaatgggc | pUC4K forward primer for +1S17 |

| IDT5593 | gtcgagcgcgagcgcatttaatcgcggcctcgagcaagacg | pUC4K reverse primer for +1S17 |
| --- | --- | --- |
| IDT5594 | aggccgcgattaaatgcgctcgcgctcgacac | DnaB1 forward primer for +1S17 |
| IDT5595 | agcatccatgttggagttgtgcgtgggaacc | DnaB1 reverse primer for +1S17 |
| IDT5486 | ggttcccacgcacaactcaggtgcgacaatctatcgattgtatggg | pUC4K forward primer for +1S36 |
| IDT5487 | gtcgagcgcgagcgcttgcccgacattatcgcgagccc | pUC4K reverse primer for +1S36 |
| IDT5488 | gataatgtcgggcaagcgctcgcgctcgacac | DnaB1 forward primer for +1S36 |
| IDT5489 | gattgtcgcacctgagttgtgcgtgggaacc | DnaB1 reverse primer for +1S36 |
| IDT5490 | ggttcccacgcacaacagcgttgccaatgatgttacagatgagatg | pUC4K forward primer for +1S60 |
| IDT5491 | gtcgagcgcgagcgcacctttgccatgtttcagaaacaactctgg | pUC4K reverse primer for +1S60 |
| IDT5492 | gaaacatggcaaaggtgcgctcgcgctcgacac | DnaB1 forward primer for +1S60 |
| IDT5493 | catcattggcaacgctgttgtgcgtgggaacc | DnaB1 reverse primer for +1S60 |
| IDT5494 | ggttcccacgcacaactcaggtgaaaatattgttgatgcgctggc | pUC4K forward primer for +1S116 |
| IDT5495 | gtcgagcgcgagcgcatcaggatattcttctaatacctggaatgctgttttcc | pUC4K reverse primer for +1S116 |

| IDT5496 | gaagaatatcctgatgcgctcgcgctcgacac | DnaB1 forward primer for +1S116 |
| --- | --- | --- |
| IDT5497 | aatattttcacctgagttgtgcgtgggaacc | DnaB1 reverse primer for +1S116 |
| IDT5498 | ggttcccacgcacaactcgattcctgtttgtaattgtccttttaacagcgatc | pUC4K forward primer for +1S133 |
| IDT5499 | gtcgagcgcgagcgcatgcaaccggcgcaggaac | pUC4K reverse primer for +1S133 |
| IDT5500 | ctgcgccggttgcatgcgctcgcgctcgacac | DnaB1 forward primer for +1S133 |
| IDT5501 | tcgattcctgtttgtgttgtgcgtgggaacc | DnaB1 reverse primer for +1S133 |
| IDT5502 | ggttcccacgcacaacagcgatcgcgtatttcgtctcgc | pUC4K forward primer for +1S143 |
| IDT5503 | gtcgagcgcgagcgcgttaaaaggacaattacaaacaggaatcgaatgcaac | pUC4K reverse primer for +1S143 |
| IDT5504 | gtaattgtccttttaacgcgctcgcgctcgacac | DnaB1 forward primer for +1S143 |
| IDT5505 | gacgaaatacgcgatcgttgtgcgtgggaacc | DnaB1 reverse primer for +1S143 |
| IDT5506 | ggttcccacgcacaactcacgaatgaataacggtttggttgatgcgag | pUC4K forward primer for +1S154 |
| IDT5507 | gtcgagcgcgagcgcttgcgcctgagcgagacgaaatac | pUC4K reverse primer for +1S154 |
| IDT5508 | ctcgctcaggcgcaagcgctcgcgctcgacac | DnaB1 forward primer for +1S154 |
| IDT5509 | gttattcattcgtgagttgtgcgtgggaacc | DnaB1 reverse primer for +1S154 |

| IDT5510 | ggttcccacgcacaacagtgattttgatgacgagcgtaatggctg | pUC4K forward primer for +1S164 |
| --- | --- | --- |
| IDT5511 | gtcgagcgcgagcgccgcatcaaccaaaccgttattcattcgtg | pUC4K reverse primer for +1S164 |
| IDT5512 | ggtttggttgatgcggcgctcgcgctcgacac | DnaB1 forward primer for +1S164 |
| IDT5513 | gtcgagcgcgagcgccgcatcaaccaaaccgttattcattcgtg | DnaBi1 reverse primer for +1S164 |
| IDT5189 | ggttcccacgcacaactcaccggattcagtcgtcactcatg | pUC4K forward primer for +1S189 |
| IDT5190 | gaatggcaaaagcttatgcatttctttccag | pUC4K reverse primer for +1S189 |
| IDT5191 | gcgctcgcgctcgac | DnaB1 forward primer for +1S189 |
| IDT5192 | gactgaatccggtgagttgtgcgtgggaaccatgc | DnaB1 reverse primer for +1S189 |
| IDT5596 | ggttcccacgcacaactcagtcgtcactcatggtgatttctcacttg | pUC4K forward primer for +1S191 |
| IDT5597 | gtcgagcgcgagcgcatccggtgagaatggcaaaagcttatgc | pUC4K reverse primer for +1S191 |
| IDT5598 | ccattctcaccggatgcgctcgcgctcgacac | DnaB1 forward primer for +1S191 |
| IDT5599 | atgagtgacgactgagttgtgcgtgggaacc | DnaB1 reverse primer for +1S191 |
| IDT5514 | ggttcccacgcacaactcacttgataaccttatttttgacgaggggaaattaatagg | pUC4K forward primer for +1S200 |

| IDT5515 | gtcgagcgcgagcgcgaaatcaccatgagtgacgactgaatccg | pUC4K reverse primer for +1S200 |
| --- | --- | --- |
| IDT5516 | actcatggtgatttcgcgctcgcgctcgacac | DnaB1 forward primer for +1S200 |
| IDT5517 | aataaggttatcaaggttgtgcgtgggaacc | DnaB1 reverse primer for +1S200 |
| IDT5518 | ggttcccacgcacaactctccttcattacagaaacggctttttcaaaaatatgg | pUC4K forward primer for +1S240 |
| IDT5519 | gtcgagcgcgagcgcaaactcaccgaggcagttccataggatg | pUC4K reverse primer for +1S240 |
| IDT5520 | tgcctcggtgagtttgcgctcgcgctcgacac | DnaB1 forward primer for +1S240 |
| IDT5521 | ctgtaatgaaggagagttgtgcgtgggaacc | DnaB1 reverse primer for +1S240 |
| IDT5600 | ggttcccacgcacaactcattacagaaacggctttttcaaaaatatggtattgataatcctg | pUC4K forward primer for +1S242 |
| IDT5601 | gtcgagcgcgagcgcaggagaaaactcaccgaggcagttcc | pUC4K reverse primer for +1S242 |
| IDT5602 | ggtgagttttctcctgcgctcgctctcgacac | DnaB1 forward primer for +1S242 |
| IDT5603 | ccgtttctgtaatgagttgtggcgtgggaacc | DnaB1 reverse primer for +1S242 |
| IDT3535 | ggggcatgcagctcgtccgggtgtgggcaaggcg | *M. leprae* DnaBi1 forward primer for MIG with SphI site |
| IDT3536 | cccatcgatcgaccgcatgaagtccagcccaagggtcga | *M. leprae* DnaBi1 reverse primer for MIG with ClaI site |

| IDT3941 | tcgagggaaggccttacatgcatgcatgctgcgcgtcccggtgtgggt | *M. smegmatis* DnaBi1 forward primer for MIG with SphI site |
| --- | --- | --- |
| IDT3942 | cttctcctttgctcatatcgatcgaccgcatgaagtccagcc | *M. smegmatis* DnaBi1 reverse primer for MIG with ClaI site |
