# Supplementary material for: Conditional DnaB Protein Splicing Is Reversibly Inhibited by Zinc in Mycobacteria
Source: mBio. 2020 Jul 14;11(4):e01403-20. doi: 10.1128/mBio.01403-20 (PMC7360933; doi:10.1128/mBio.01403-20)
Supplement: TABLE S3 [file mBio.01403-20-st003.docx]

**Supplemental Table 3.** Raw CFU data for KanR, KanS, and KD fusions with mean of 3 independent experiments ± standard deviation.

A. Kanamycin controls (KanR and KanS)

| [kan]μg/ml | KanR  n=3 | | | KanR  mean ±s.d. | | KanS  n=3 | | | KanS  mean ±s.d. | |
| --- | --- | --- | --- | --- | --- | --- | --- | --- | --- | --- |
| 0 | 5×10^7^ | 7×10^7^ | 1×10^8^ | 7.33×10^7^ | 2.52×10^7^ | 2×10^8^ | 1×10^8^ | 3×10^8^ | 2×10^8^ | 1×10^8^ |
| 12.5 | 4×10^7^ | 1×10^8^ | 8×10^7^ | 7.33×10^7^ | 3.06×10^7^ | 0 | 0 | 0 | 0 | 0 |
| 25 | 8×10^7^ | 8×10^7^ | 6×10^7^ | 7.33×10^7^ | 1.15×10^7^ | 0 | 0 | 0 | 0 | 0 |
| 50 | 5×10^7^ | 7×10^7^ | 7×10^7^ | 6.33×10^7^ | 1.15×10^7^ | 0 | 0 | 0 | 0 | 0 |
| 75 | 8×10^7^ | 1×10^8^ | 8×10^7^ | 8.67×10^7^ | 1.15×10^7^ | 0 | 0 | 0 | 0 | 0 |
| 100 | 5×10^7^ | 8×10^7^ | 7×10^7^ | 6.67×10^7^ | 1.53×10^7^ | 0 | 0 | 0 | 0 | 0 |
| 150 | 7×10^7^ | 8×10^7^ | 8×10^7^ | 7.67×10^7^ | 5.77×10^6^ | 0 | 0 | 0 | 0 | 0 |
| 200 | 6×10^7^ | 7×10^7^ | 7×10^7^ | 6.67×10^7^ | 5.77×10^6^ | 0 | 0 | 0 | 0 | 0 |
| 250 | 7×10^7^ | 7×10^7^ | 8×10^7^ | 7.33×10^7^ | 5.77×10^6^ | 0 | 0 | 0 | 0 | 0 |
| 300 | 7×10^7^ | 1×10^8^ | 8×10^7^ | 8.33×10^7^ | 1.53×10^7^ | 0 | 0 | 0 | 0 | 0 |
| 400 | 4×10^7^ | 8×10^7^ | 6×10^7^ | 6.00×10^7^ | 2.00×10^7^ | 0 | 0 | 0 | 0 | 0 |
| 500 | 5×10^7^ | 5×10^7^ | 6×10^7^ | 5.33×10^7^ | 5.77×10^6^ | 0 | 0 | 0 | 0 | 0 |
| 750 | 2×10^7^ | 5×10^7^ | 4×10^7^ | 3.67×10^7^ | 1.53×10^7^ | 0 | 0 | 0 | 0 | 0 |
| 1000 | 4×10^7^ | 8×10^7^ | 7×10^7^ | 6.33×10^7^ | 2.08×10^7^ | 0 | 0 | 0 | 0 | 0 |
| 1500 | 2×10^7^ | 5×10^7^ | 5×10^7^ | 4.00×10^7^ | 1.73×10^7^ | 0 | 0 | 0 | 0 | 0 |
| 2000 | 4×10^7^ | 3×10^7^ | 7×10^7^ | 4.67×10^7^ | 2.08×10^7^ | 0 | 0 | 0 | 0 | 0 |

B. Splicing-dependent resistance (SDR): KD36

| [kan]μg/ml | KD36WT  n=3 | | | KD36WT  mean ±s.d. | | KD36^C118A^  n=3 | | | KD36^C118A^  mean ±s.d. | |
| --- | --- | --- | --- | --- | --- | --- | --- | --- | --- | --- |
| 0 | 6×10^7^ | 6×10^7^ | 8×10^7^ | 6.67×10^7^ | 2.86×10^7^ | 2×10^8^ | 8×10^7^ | 8×10^7^ | 1.20×10^8^ | 6.93×10^7^ |
| 12.5 | 1×10^7^ | 2×10^7^ | 2×10^7^ | 1.67×10^7^ | 1.64×10^7^ | 0 | 0 | 0 | 0 | 0 |
| 25 | 1×10^7^ | 5×10^7^ | 4×10^7^ | 3.33×10^7^ | 1.60×10^7^ | 0 | 0 | 0 | 0 | 0 |
| 50 | 1×10^7^ | 3×10^7^ | 3×10^7^ | 2.33×10^7^ | 1.47×10^7^ | 0 | 0 | 0 | 0 | 0 |
| 75 | 3×10^4^ | 2×10^4^ | 4×10^7^ | 3.00×10^4^ | 1.74×10^4^ | 0 | 0 | 0 | 0 | 0 |
| 100 | 1×10^3^ | 0 | 0 | 3.33×10^2^ | 3.33×10^2^ | 0 | 0 | 0 | 0 | 0 |
| 150 | 0 | 0 | 0 | 0 | 0 | 0 | 0 | 0 | 0 | 0 |
| 200 | 0 | 0 | 0 | 0 | 0 | 0 | 0 | 0 | 0 | 0 |
| 250 | 0 | 0 | 0 | 0 | 0 | 0 | 0 | 0 | 0 | 0 |
| 300 | 0 | 0 | 0 | 0 | 0 | 0 | 0 | 0 | 0 | 0 |
| 400 | 0 | 0 | 0 | 0 | 0 | 0 | 0 | 0 | 0 | 0 |
| 500 | 0 | 0 | 0 | 0 | 0 | 0 | 0 | 0 | 0 | 0 |
| 750 | 0 | 0 | 0 | 0 | 0 | 0 | 0 | 0 | 0 | 0 |
| 1000 | 0 | 0 | 0 | 0 | 0 | 0 | 0 | 0 | 0 | 0 |
| 1500 | 0 | 0 | 0 | 0 | 0 | 0 | 0 | 0 | 0 | 0 |
| 2000 | 0 | 0 | 0 | 0 | 0 | 0 | 0 | 0 | 0 | 0 |

C. Splicing-dependent resistance (SDR): KD60

| [kan]μg/ml | KD60WT  n=3 | | | KD60WT  mean ±s.d. | | KD60^C118A^  n=3 | | | KD60^C118A^  mean ±s.d. | |
| --- | --- | --- | --- | --- | --- | --- | --- | --- | --- | --- |
| 0 | 8×10^7^ | 8×10^7^ | 1×10^8^ | 8.67×10^7^ | 1.15×10^7^ | 9×10^7^ | 8×10^7^ | 9×10^7^ | 8.67×10^7^ | 5.77×10^6^ |
| 12.5 | 5×10^7^ | 7×10^7^ | 5×10^7^ | 5.67×10^7^ | 1.15×10^7^ | 0 | 0 | 0 | 0 | 0 |
| 25 | 1×10^5^ | 8×10^4^ | 2×10^5^ | 1.27×10^5^ | 6.43×10^4^ | 0 | 0 | 0 | 0 | 0 |
| 50 | 5×10^4^ | 6×10^4^ | 6×10^4^ | 5.67×10^4^ | 5.77×10^3^ | 0 | 0 | 0 | 0 | 0 |
| 75 | 3×10^3^ | 2×10^3^ | 3×10^3^ | 2.67×10^3^ | 5.77×10^2^ | 0 | 0 | 0 | 0 | 0 |
| 100 | 0 | 0 | 0 | 0 | 0 | 0 | 0 | 0 | 0 | 0 |
| 150 | 0 | 0 | 0 | 0 | 0 | 0 | 0 | 0 | 0 | 0 |
| 200 | 0 | 0 | 0 | 0 | 0 | 0 | 0 | 0 | 0 | 0 |
| 250 | 0 | 0 | 0 | 0 | 0 | 0 | 0 | 0 | 0 | 0 |
| 300 | 0 | 0 | 0 | 0 | 0 | 0 | 0 | 0 | 0 | 0 |
| 400 | 0 | 0 | 0 | 0 | 0 | 0 | 0 | 0 | 0 | 0 |
| 500 | 0 | 0 | 0 | 0 | 0 | 0 | 0 | 0 | 0 | 0 |
| 750 | 0 | 0 | 0 | 0 | 0 | 0 | 0 | 0 | 0 | 0 |
| 1000 | 0 | 0 | 0 | 0 | 0 | 0 | 0 | 0 | 0 | 0 |
| 1500 | 0 | 0 | 0 | 0 | 0 | 0 | 0 | 0 | 0 | 0 |
| 2000 | 0 | 0 | 0 | 0 | 0 | 0 | 0 | 0 | 0 | 0 |

D. Splicing-dependent resistance (SDR): KD154

| [kan]μg/ml | KD154WT  n=3 | | | KD154WT  mean ±s.d. | | KD154^C118A^  n=3 | | | KD154^C118A^  mean ±s.d. | |
| --- | --- | --- | --- | --- | --- | --- | --- | --- | --- | --- |
| 0 | 1×10^8^ | 1×10^8^ | 9×10^7^ | 9.67×10^7^ | 5.77×10^6^ | 5×10^7^ | 6×10^7^ | 6×10^7^ | 5.67×10^7^ | 5.77×10^6^ |
| 12.5 | 6×10^7^ | 6×10^7^ | 7×10^7^ | 6.33×10^7^ | 5.77×10^6^ | 0 | 0 | 0 | 0 | 0 |
| 25 | 6×10^7^ | 5×10^7^ | 6×10^7^ | 5.67×10^7^ | 5.77×10^6^ | 0 | 0 | 0 | 0 | 0 |
| 50 | 4×10^7^ | 5×10^7^ | 6×10^7^ | 5.00×10^7^ | 1.00×10^7^ | 0 | 0 | 0 | 0 | 0 |
| 75 | 6×10^7^ | 6×10^7^ | 7×10^7^ | 6.33×10^7^ | 5.77×10^6^ | 0 | 0 | 0 | 0 | 0 |
| 100 | 5×10^7^ | 7×10^7^ | 6×10^7^ | 6.00×10^7^ | 1.00×10^7^ | 0 | 0 | 0 | 0 | 0 |
| 150 | 3×10^7^ | 6×10^7^ | 5×10^7^ | 4.67×10^7^ | 1.53×10^7^ | 0 | 0 | 0 | 0 | 0 |
| 200 | 8×10^5^ | 5×10^5^ | 1×10^6^ | 7.67×10^5^ | 2.52×10^5^ | 0 | 0 | 0 | 0 | 0 |
| 250 | 3×10^3^ | 2×10^3^ | 3×10^3^ | 2.67×10^3^ | 5.77×10^2^ | 0 | 0 | 0 | 0 | 0 |
| 300 | 4×10^3^ | 3×10^3^ | 3×10^3^ | 3.33×10^3^ | 5.77×10^2^ | 0 | 0 | 0 | 0 | 0 |
| 400 | 4×10^3^ | 1×10^3^ | 3×10^3^ | 2.67×10^3^ | 1.53×10^3^ | 0 | 0 | 0 | 0 | 0 |
| 500 | 0 | 0 | 0 | 0 | 0 | 0 | 0 | 0 | 0 | 0 |
| 750 | 0 | 0 | 0 | 0 | 0 | 0 | 0 | 0 | 0 | 0 |
| 1000 | 0 | 0 | 0 | 0 | 0 | 0 | 0 | 0 | 0 | 0 |
| 1500 | 0 | 0 | 0 | 0 | 0 | 0 | 0 | 0 | 0 | 0 |
| 2000 | 0 | 0 | 0 | 0 | 0 | 0 | 0 | 0 | 0 | 0 |

E. Splicing-dependent resistance (SDR): KD164

| [kan]μg/ml | KD164WT  n=3 | | | KD164WT  mean ±s.d. | | KD164^C118A^  n=3 | | | KD164^C118A^  mean ±s.d. | |
| --- | --- | --- | --- | --- | --- | --- | --- | --- | --- | --- |
| 0 | 4×10^7^ | 6×10^7^ | 7×10^7^ | 5.67×10^7^ | 1.53×10^7^ | 5×10^6^ | 4×10^6^ | 5×10^6^ | 4.67×10^6^ | 5.77×10^5^ |
| 12.5 | 3×10^7^ | 4×10^7^ | 3×10^7^ | 3.33×10^7^ | 5.77×10^6^ | 0 | 0 | 0 | 0 | 0 |
| 25 | 1×10^7^ | 9×10^6^ | 2×10^7^ | 1.30×10^7^ | 6.08×10^6^ | 0 | 0 | 0 | 0 | 0 |
| 50 | 8×10^6^ | 9×10^6^ | 1×10^7^ | 9.00×10^6^ | 1.00×10^6^ | 0 | 0 | 0 | 0 | 0 |
| 75 | 1×10^5^ | 9×10^4^ | 2×10^5^ | 1.30×10^5^ | 6.08×10^4^ | 0 | 0 | 0 | 0 | 0 |
| 100 | 1×10^3^ | 2×10^3^ | 1×10^3^ | 1.33×10^3^ | 5.77×10^2^ | 0 | 0 | 0 | 0 | 0 |
| 150 | 0 | 0 | 0 | 0 | 0 | 0 | 0 | 0 | 0 | 0 |
| 200 | 0 | 0 | 0 | 0 | 0 | 0 | 0 | 0 | 0 | 0 |
| 250 | 0 | 0 | 0 | 0 | 0 | 0 | 0 | 0 | 0 | 0 |
| 300 | 0 | 0 | 0 | 0 | 0 | 0 | 0 | 0 | 0 | 0 |
| 400 | 0 | 0 | 0 | 0 | 0 | 0 | 0 | 0 | 0 | 0 |
| 500 | 0 | 0 | 0 | 0 | 0 | 0 | 0 | 0 | 0 | 0 |
| 750 | 0 | 0 | 0 | 0 | 0 | 0 | 0 | 0 | 0 | 0 |
| 1000 | 0 | 0 | 0 | 0 | 0 | 0 | 0 | 0 | 0 | 0 |
| 1500 | 0 | 0 | 0 | 0 | 0 | 0 | 0 | 0 | 0 | 0 |
| 2000 | 0 | 0 | 0 | 0 | 0 | 0 | 0 | 0 | 0 | 0 |

F. Splicing-dependent resistance (SDR): KD200

| [kan]μg/ml | KD200WT  n=3 | | | KD200WT  mean ±s.d. | | KD200^C118A^  n=3 | | | KD200^C118A^  mean ±s.d. | |
| --- | --- | --- | --- | --- | --- | --- | --- | --- | --- | --- |
| 0 | 4×10^7^ | 6×10^7^ | 6×10^7^ | 5.33×10^7^ | 1.15×10^7^ | 2×10^7^ | 3×10^7^ | 3×10^7^ | 2.67×10^7^ | 5.77×10^6^ |
| 12.5 | 4×10^4^ | 3×10^4^ | 4×10^4^ | 3.67×10^4^ | 5.77×10^3^ | 0 | 0 | 0 | 0 | 0 |
| 25 | 3×10^4^ | 3×10^4^ | 3×10^4^ | 3.00×10^4^ | 0 | 0 | 0 | 0 | 0 | 0 |
| 50 | 0 | 0 | 0 | 0 | 0 | 0 | 0 | 0 | 0 | 0 |
| 75 | 0 | 0 | 0 | 0 | 0 | 0 | 0 | 0 | 0 | 0 |
| 100 | 0 | 0 | 0 | 0 | 0 | 0 | 0 | 0 | 0 | 0 |
| 150 | 0 | 0 | 0 | 0 | 0 | 0 | 0 | 0 | 0 | 0 |
| 200 | 0 | 0 | 0 | 0 | 0 | 0 | 0 | 0 | 0 | 0 |
| 250 | 0 | 0 | 0 | 0 | 0 | 0 | 0 | 0 | 0 | 0 |
| 300 | 0 | 0 | 0 | 0 | 0 | 0 | 0 | 0 | 0 | 0 |
| 400 | 0 | 0 | 0 | 0 | 0 | 0 | 0 | 0 | 0 | 0 |
| 500 | 0 | 0 | 0 | 0 | 0 | 0 | 0 | 0 | 0 | 0 |
| 750 | 0 | 0 | 0 | 0 | 0 | 0 | 0 | 0 | 0 | 0 |
| 1000 | 0 | 0 | 0 | 0 | 0 | 0 | 0 | 0 | 0 | 0 |
| 1500 | 0 | 0 | 0 | 0 | 0 | 0 | 0 | 0 | 0 | 0 |
| 2000 | 0 | 0 | 0 | 0 | 0 | 0 | 0 | 0 | 0 | 0 |

G. Splicing-independent resistance (SIR): KD2

| [kan]μg/ml | KD2WT  n=3 | | | KD2WT  mean ±s.d. | | KD2^C118A^  n=3 | | | KD2^C118A^  mean ±s.d. | |
| --- | --- | --- | --- | --- | --- | --- | --- | --- | --- | --- |
| 0 | 3×10^8^ | 2×10^8^ | 3×10^8^ | 2.67×10^8^ | 5.77×10^7^ | 4×10^8^ | 3×10^8^ | 3×10^8^ | 3.33×10^8^ | 5.77×10^7^ |
| 12.5 | 4×10^8^ | 3×10^8^ | 3×10^8^ | 3.33×10^8^ | 5.77×10^7^ | 1×10^8^ | 9×10^7^ | 2×10^8^ | 1.30×10^8^ | 6.08×10^7^ |
| 25 | 2×10^8^ | 3×10^8^ | 9×10^7^ | 1.97×10^8^ | 1.05×10^8^ | 9×10^7^ | 8×10^7^ | 9×10^7^ | 8.67×10^7^ | 5.77×10^6^ |
| 50 | 9×10^7^ | 1×10^8^ | 1×10^8^ | 9.67×10^7^ | 5.77×10^6^ | 8×10^7^ | 7×10^7^ | 7×10^7^ | 7.33×10^7^ | 5.77×10^6^ |
| 75 | 3×10^7^ | 1×10^7^ | 9×10^8^ | 1.63×10^7^ | 1.18×10^7^ | 2×10^6^ | 3×10^6^ | 3×10^6^ | 2.67×10^6^ | 5.77×10^5^ |
| 100 | 9×10^6^ | 8×10^6^ | 8×10^8^ | 8.33×10^6^ | 5.77×10^5^ | 2×10^6^ | 1×10^6^ | 1×10^6^ | 1.33×10^6^ | 5.77×10^5^ |
| 150 | 5×10^6^ | 4×10^6^ | 5×10^8^ | 4.67×10^6^ | 5.77×10^5^ | 8×10^5^ | 7×10^5^ | 7×10^5^ | 7.33×10^5^ | 5.77×10^4^ |
| 200 | 3×10^6^ | 3×10^6^ | 4×10^8^ | 3.33×10^6^ | 5.77×10^5^ | 4×10^5^ | 2×10^5^ | 2×10^5^ | 2.67×10^5^ | 1.15×10^5^ |
| 250 | 7×10^5^ | 1×10^6^ | 9×10^8^ | 8.67×10^5^ | 1.53×10^5^ | 8×10^4^ | 1×10^5^ | 9×10^4^ | 9.00×10^4^ | 1.00×10^4^ |
| 300 | 4×10^5^ | 5×10^5^ | 5×10^8^ | 4.67×10^5^ | 5.77×10^4^ | 5×10^4^ | 3×10^4^ | 4×10^4^ | 4.00×10^4^ | 1.00×10^4^ |
| 400 | 9×10^4^ | 7×10^4^ | 1×10^8^ | 8.67×10^4^ | 1.53×10^4^ | 0 | 0 | 0 | 0 | 0 |
| 500 | 0 | 0 | 0 | 0 | 0 | 0 | 0 | 0 | 0 | 0 |
| 750 | 0 | 0 | 0 | 0 | 0 | 0 | 0 | 0 | 0 | 0 |
| 1000 | 0 | 0 | 0 | 0 | 0 | 0 | 0 | 0 | 0 | 0 |
| 1500 | 0 | 0 | 0 | 0 | 0 | 0 | 0 | 0 | 0 | 0 |
| 2000 | 0 | 0 | 0 | 0 | 0 | 0 | 0 | 0 | 0 | 0 |

H. Splicing-independent resistance (SIR): KD9

| [kan]μg/ml | KD9WT  n=3 | | | KD9WT  mean ±s.d. | | KD9^C118A^  n=3 | | | KD9^C118A^  mean ±s.d. | |
| --- | --- | --- | --- | --- | --- | --- | --- | --- | --- | --- |
| 0 | 3×10^8^ | 3×10^8^ | 2×10^8^ | 2.67×10^8^ | 5.77×10^7^ | 1×10^8^ | 2×10^8^ | 3×10^8^ | 2.00×10^8^ | 1.00×10^8^ |
| 12.5 | 2×10^8^ | 2×10^8^ | 2×10^8^ | 2.00×10^8^ | 0 | 3×10^8^ | 3×10^8^ | 2×10^8^ | 2.67×10^8^ | 5.77×10^7^ |
| 25 | 4×10^8^ | 3×10^8^ | 2×10^8^ | 3.00×10^8^ | 1.00×10^8^ | 4×10^8^ | 3×10^8^ | 2×10^8^ | 3.00×10^8^ | 1.00×10^8^ |
| 50 | 4×10^8^ | 3×10^8^ | 3×10^8^ | 3.33×10^8^ | 5.77×10^7^ | 4×10^8^ | 1×10^8^ | 2×10^8^ | 2.33×10^8^ | 1.53×10^8^ |
| 75 | 4×10^8^ | 4×10^8^ | 3×10^8^ | 3.67×10^8^ | 5.77×10^7^ | 8×10^7^ | 7×10^7^ | 9×10^7^ | 8.00×10^7^ | 1.00×10^7^ |
| 100 | 3×10^8^ | 3×10^8^ | 3×10^8^ | 3.00×10^8^ | 0 | 3×10^8^ | 1×10^8^ | 3×10^8^ | 2.33×10^8^ | 1.15×10^8^ |
| 150 | 2×10^8^ | 1×10^8^ | 2×10^8^ | 1.67×10^8^ | 5.77×10^7^ | 5×10^8^ | 4×10^8^ | 4×10^8^ | 4.33×10^8^ | 5.77×10^7^ |
| 200 | 2×10^8^ | 3×10^8^ | 1×10^8^ | 2.00×10^8^ | 1.00×10^8^ | 3×10^8^ | 3×10^8^ | 2×10^8^ | 2.67×10^7^ | 5.77×10^7^ |
| 250 | 1×10^8^ | 9×10^7^ | 1×10^8^ | 9.67×10^7^ | 5.77×10^6^ | 1×10^8^ | 9×10^7^ | 1×10^8^ | 9.67×10^8^ | 5.77×10^6^ |
| 300 | 4×10^8^ | 3×10^8^ | 3×10^8^ | 3.33×10^8^ | 5.77×10^7^ | 4×10^8^ | 3×10^8^ | 4×10^8^ | 3.67×10^8^ | 5.77×10^7^ |
| 400 | 6×10^8^ | 4×10^8^ | 2×10^8^ | 4.00×10^8^ | 2.00×10^8^ | 4×10^8^ | 4×10^8^ | 2×10^8^ | 3.33×10^8^ | 1.15×10^8^ |
| 500 | 6×10^8^ | 2×10^8^ | 3×10^8^ | 3.67×10^8^ | 2.08×10^8^ | 4×10^8^ | 3×10^8^ | 3×10^8^ | 3.33×10^8^ | 5.77×10^7^ |
| 750 | 6×10^7^ | 5×10^7^ | 6×10^7^ | 5.67×10^7^ | 5.77×10^6^ | 5×10^7^ | 6×10^7^ | 3×10^7^ | 4.67×10^7^ | 1.53×10^7^ |
| 1000 | 6×10^4^ | 8×10^4^ | 5×10^4^ | 6.33×10^4^ | 1.53×10^4^ | 2×10^5^ | 5×10^6^ | 8×10^5^ | 2.00×10^6^ | 2.62×10^6^ |
| 1500 | 3×10^3^ | 3×10^3^ | 1×10^3^ | 2.33×10^3^ | 1.15×10^3^ | 4×10^3^ | 5×10^3^ | 4×10^3^ | 4.33×10^3^ | 5.77×10^2^ |
| 2000 | 0 | 0 | 0 | 0 | 0 | 0 | 0 | 0 | 0 | 0 |

I. Splicing-independent resistance (SIR): KD11

| [kan]μg/ml | KD11WT  n=3 | | | KD11WT  mean ±s.d. | | KD11^C118A^  n=3 | | | KD11^C118A^  mean ±s.d. | |
| --- | --- | --- | --- | --- | --- | --- | --- | --- | --- | --- |
| 0 | 4×10^7^ | 1×10^8^ | 9×10^7^ | 7.67×10^7^ | 3.21×10^7^ | 5×10^7^ | 8×10^7^ | 6×10^7^ | 6.33×10^7^ | 1.53×10^7^ |
| 12.5 | 3×10^7^ | 4×10^7^ | 4×10^7^ | 3.67×10^7^ | 5.77×10^6^ | 1×10^8^ | 9×10^7^ | 1×10^8^ | 9.67×10^7^ | 5.77×10^6^ |
| 25 | 2×10^8^ | 8×10^7^ | 1×10^8^ | 1.27×10^8^ | 6.43×10^7^ | 8×10^7^ | 9×10^7^ | 1×10^8^ | 9.00×10^7^ | 1.00×10^7^ |
| 50 | 2×10^8^ | 1×10^8^ | 9×10^7^ | 1.30×10^8^ | 6.08×10^7^ | 8×10^7^ | 1×10^8^ | 9×10^7^ | 9.00×10^7^ | 1.00×10^7^ |
| 75 | 7×10^7^ | 5×10^7^ | 7×10^7^ | 6.33×10^7^ | 1.15×10^7^ | 2×10^8^ | 3×10^8^ | 2×10^8^ | 2.33×10^8^ | 5.77×10^7^ |
| 100 | 3×10^7^ | 4×10^7^ | 4×10^7^ | 3.67×10^7^ | 5.77×10^6^ | 3×10^8^ | 2×10^8^ | 1×10^8^ | 2.00×10^8^ | 1.00×10^8^ |
| 150 | 4×10^7^ | 5×10^7^ | 3×10^7^ | 4.00×10^7^ | 1.00×10^7^ | 4×10^7^ | 6×10^7^ | 7×10^7^ | 5.67×10^7^ | 1.53×10^7^ |
| 200 | 4×10^7^ | 6×10^7^ | 5×10^7^ | 5.00×10^7^ | 1.00×10^7^ | 2×10^7^ | 1×10^7^ | 4×10^7^ | 2.33×10^7^ | 1.53×10^7^ |
| 250 | 4×10^7^ | 4×10^7^ | 4×10^7^ | 4.00×10^7^ | 0 | 4×10^7^ | 2×10^7^ | 2×10^7^ | 2.67×10^7^ | 1.15×10^7^ |
| 300 | 1×10^8^ | 8×10^7^ | 7×10^7^ | 8.33×10^7^ | 1.53×10^7^ | 3×10^8^ | 4×10^8^ | 3×10^8^ | 3.33×10^8^ | 5.77×10^7^ |
| 400 | 3×10^7^ | 5×10^7^ | 3×10^7^ | 3.67×10^7^ | 1.15×10^7^ | 1×10^8^ | 9×10^7^ | 1×10^8^ | 9.67×10^8^ | 5.77×10^6^ |
| 500 | 5×10^7^ | 4×10^7^ | 5×10^7^ | 4.67×10^7^ | 5.77×10^6^ | 4×10^7^ | 3×10^7^ | 3×10^7^ | 3.33×10^7^ | 5.77×10^6^ |
| 750 | 8×10^6^ | 6×10^6^ | 8×10^6^ | 7.33×10^6^ | 1.15×10^6^ | 3×10^7^ | 2×10^7^ | 4×10^7^ | 3.00×10^7^ | 1.00×10^7^ |
| 1000 | 2×10^7^ | 8×10^6^ | 1×10^7^ | 1.27×10^7^ | 6.43×10^6^ | 2×10^7^ | 1×10^7^ | 8×10^6^ | 1.27×10^6^ | 6.43×10^6^ |
| 1500 | 2×10^5^ | 3×10^5^ | 2×10^5^ | 2.33×10^5^ | 5.77×10^4^ | 1×10^4^ | 2×10^4^ | 8×10^3^ | 1.27×10^3^ | 6.43×10^3^ |
| 2000 | 0 | 0 | 0 | 0 | 0 | 2×10^3^ | 1×10^3^ | 2×10^3^ | 1.67×10^3^ | 5.77×10^2^ |

J. Splicing-independent resistance (SIR): KD17

| [kan]μg/ml | KD17WT  n=3 | | | KD17WT  mean ±s.d. | | KD17^C118A^  n=3 | | | KD17^C118A^  mean ±s.d. | |
| --- | --- | --- | --- | --- | --- | --- | --- | --- | --- | --- |
| 0 | 3×10^7^ | 4×10^7^ | 6×10^7^ | 4.33×10^7^ | 1.53×10^7^ | 4×10^7^ | 5×10^7^ | 6×10^7^ | 5.00×10^7^ | 1.00×10^7^ |
| 12.5 | 5×10^7^ | 4×10^7^ | 5×10^7^ | 4.67×10^7^ | 5.77×10^6^ | 2×10^7^ | 2×10^7^ | 3×10^7^ | 2.33×10^7^ | 5.77×10^6^ |
| 25 | 8×10^7^ | 6×10^7^ | 7×10^7^ | 7.00×10^7^ | 1.00×10^7^ | 8×10^7^ | 5×10^7^ | 8×10^7^ | 7.00×10^7^ | 1.73×10^7^ |
| 50 | 7×10^7^ | 7×10^7^ | 8×10^7^ | 7.33×10^7^ | 5.77×10^6^ | 7×10^7^ | 7×10^7^ | 7×10^7^ | 7.00×10^7^ | 0 |
| 75 | 7×10^7^ | 7×10^7^ | 7×10^7^ | 7.00×10^7^ | 0 | 7×10^7^ | 6×10^7^ | 5×10^7^ | 6.00×10^7^ | 1.00×10^7^ |
| 100 | 1×10^8^ | 8×10^7^ | 8×10^7^ | 8.67×10^7^ | 1.15×10^7^ | 8×10^7^ | 7×10^7^ | 7×10^7^ | 7.33×10^7^ | 5.77×10^6^ |
| 150 | 2×10^8^ | 1×10^8^ | 7×10^7^ | 1.23×10^8^ | 6.81×10^7^ | 6×10^7^ | 8×10^7^ | 6×10^7^ | 6.67×10^7^ | 1.15×10^7^ |
| 200 | 8×10^7^ | 7×10^7^ | 8×10^7^ | 7.67×10^7^ | 5.77×10^6^ | 5×10^7^ | 5×10^7^ | 4×10^7^ | 4.67×10^7^ | 5.77×10^6^ |
| 250 | 3×10^7^ | 4×10^7^ | 2×10^7^ | 3.00×10^7^ | 1.00×10^7^ | 3×10^7^ | 5×10^7^ | 3×10^7^ | 3.67×10^7^ | 1.15×10^7^ |
| 300 | 4×10^7^ | 4×10^7^ | 3×10^7^ | 3.67×10^7^ | 5.77×10^6^ | 4×10^7^ | 9×10^6^ | 5×10^7^ | 3.30×10^7^ | 2.14×10^7^ |
| 400 | 5×10^7^ | 5×10^7^ | 7×10^7^ | 5.67×10^7^ | 1.15×10^7^ | 4×10^7^ | 4×10^7^ | 3×10^7^ | 3.67×10^7^ | 5.77×10^6^ |
| 500 | 5×10^7^ | 4×10^7^ | 2×10^7^ | 3.67×10^7^ | 1.53×10^7^ | 5×10^7^ | 5×10^7^ | 5×10^7^ | 5.00×10^7^ | 0 |
| 750 | 2×10^7^ | 1×10^7^ | 9×10^6^ | 1.30×10^7^ | 6.08×10^6^ | 9×10^6^ | 1×10^7^ | 9×10^6^ | 9.33×10^6^ | 5.77×10^5^ |
| 1000 | 3×10^7^ | 9×10^6^ | 2×10^7^ | 1.97×10^7^ | 1.05×10^7^ | 8×10^6^ | 5×10^6^ | 8×10^6^ | 7.00×10^6^ | 1.73×10^6^ |
| 1500 | 1×10^4^ | 1×10^4^ | 9×10^3^ | 9.67×10^3^ | 5.77×10^2^ | 5×10^4^ | 3×10^4^ | 4×10^4^ | 4.00×10^4^ | 1.00×10^4^ |
| 2000 | 0 | 0 | 0 | 0 | 0 | 2×10^3^ | 2×10^3^ | 1×10^3^ | 1.67×10^3^ | 5.77×10^2^ |

K. Splicing-independent resistance (SIR): KD116

| [kan]μg/ml | KD116WT  n=3 | | | KD116WT  mean ±s.d. | | KD116^C118A^  n=3 | | | KD116^C118A^  mean ±s.d. | |
| --- | --- | --- | --- | --- | --- | --- | --- | --- | --- | --- |
| 0 | 9×10^7^ | 8×10^7^ | 1×10^8^ | 9.00×10^7^ | 1.00×10^7^ | 1×10^8^ | 9×10^7^ | 1×10^8^ | 9.67×10^7^ | 5.77×10^6^ |
| 12.5 | 5×10^7^ | 7×10^7^ | 5×10^7^ | 5.67×10^7^ | 1.15×10^7^ | 5×10^7^ | 8×10^7^ | 6×10^7^ | 6.33×10^7^ | 1.53×10^7^ |
| 25 | 7×10^7^ | 7×10^7^ | 6×10^7^ | 6.67×10^7^ | 5.77×10^6^ | 5×10^7^ | 5×10^7^ | 6×10^7^ | 5.33×10^7^ | 5.77×10^6^ |
| 50 | 3×10^8^ | 2×10^8^ | 9×10^7^ | 1.97×10^8^ | 1.05×10^8^ | 3×10^7^ | 3×10^7^ | 6×10^7^ | 4.00×10^7^ | 1.73×10^7^ |
| 75 | 3×10^7^ | 5×10^7^ | 3×10^7^ | 3.67×10^7^ | 1.15×10^7^ | 3×10^7^ | 3×10^7^ | 4×10^7^ | 3.33×10^7^ | 5.77×10^6^ |
| 100 | 2×10^7^ | 4×10^7^ | 2×10^7^ | 2.67×10^7^ | 1.15×10^7^ | 5×10^7^ | 5×10^7^ | 5×10^7^ | 5.00×10^7^ | 0 |
| 150 | 1×10^4^ | 3×10^4^ | 3×10^4^ | 2.33×10^4^ | 1.15×10^4^ | 4×10^5^ | 5×10^5^ | 2×10^5^ | 3.67×10^5^ | 1.53×10^5^ |
| 200 | 3×10^3^ | 2×10^3^ | 1×10^3^ | 2.00×10^3^ | 1.00×10^3^ | 4×10^4^ | 3×10^4^ | 6×10^4^ | 4.33×10^4^ | 1.53×10^4^ |
| 250 | 0 | 0 | 0 | 0 | 0 | 0 | 0 | 0 | 0 | 0 |
| 300 | 0 | 0 | 0 | 0 | 0 | 0 | 0 | 0 | 0 | 0 |
| 400 | 0 | 0 | 0 | 0 | 0 | 0 | 0 | 0 | 0 | 0 |
| 500 | 0 | 0 | 0 | 0 | 0 | 0 | 0 | 0 | 0 | 0 |
| 750 | 0 | 0 | 0 | 0 | 0 | 0 | 0 | 0 | 0 | 0 |
| 1000 | 0 | 0 | 0 | 0 | 0 | 0 | 0 | 0 | 0 | 0 |
| 1500 | 0 | 0 | 0 | 0 | 0 | 0 | 0 | 0 | 0 | 0 |
| 2000 | 0 | 0 | 0 | 0 | 0 | 0 | 0 | 0 | 0 | 0 |

L. Splicing-independent resistance (SIR): KD189,

| [kan]μg/ml | KD189WT  n=3 | | | KD189WT  mean ±s.d. | | KD189^C118A^  n=3 | | | KD189^C118A^  mean ±s.d. | |
| --- | --- | --- | --- | --- | --- | --- | --- | --- | --- | --- |
| 0 | 6×10^7^ | 1×10^8^ | 8×10^7^ | 8.00×10^7^ | 2.00×10^7^ | 2×10^8^ | 1×10^8^ | 9×10^7^ | 1.30×10^8^ | 6.08×10^7^ |
| 12.5 | 6×10^7^ | 8×10^7^ | 8×10^7^ | 7.33×10^7^ | 1.15×10^7^ | 1×10^8^ | 1×10^8^ | 2×10^8^ | 1.33×10^8^ | 5.77×10^7^ |
| 25 | 3×10^7^ | 5×10^7^ | 3×10^7^ | 3.67×10^7^ | 1.15×10^7^ | 1×10^8^ | 1×10^8^ | 1×10^8^ | 1.00×10^8^ | 0 |
| 50 | 6×10^7^ | 5×10^7^ | 6×10^7^ | 5.67×10^7^ | 5.77×10^6^ | 1×10^8^ | 2×10^8^ | 9×10^7^ | 1.30×10^8^ | 6.08×10^7^ |
| 75 | 4×10^7^ | 6×10^7^ | 4×10^7^ | 4.67×10^7^ | 1.15×10^7^ | 2×10^8^ | 1×10^8^ | 1×10^8^ | 1.33×10^8^ | 5.77×10^7^ |
| 100 | 5×10^7^ | 5×10^7^ | 6×10^7^ | 5.33×10^7^ | 5.77×10^6^ | 2×10^8^ | 9×10^7^ | 9×10^7^ | 1.27×10^8^ | 6.35×10^7^ |
| 150 | 7×10^7^ | 6×10^7^ | 5×10^7^ | 6.00×10^7^ | 1.00×10^7^ | 2×10^8^ | 1×10^8^ | 2×10^8^ | 1.67×10^8^ | 5.77×10^7^ |
| 200 | 2×10^7^ | 5×10^7^ | 5×10^7^ | 4.00×10^7^ | 1.73×10^7^ | 1×10^8^ | 9×10^7^ | 1×10^8^ | 9.67×10^7^ | 5.77×10^6^ |
| 250 | 4×10^7^ | 4×10^7^ | 5×10^7^ | 4.33×10^7^ | 5.77×10^6^ | 2×10^8^ | 1×10^8^ | 9×10^7^ | 1.30×10^8^ | 6.08×10^7^ |
| 300 | 5×10^7^ | 3×10^7^ | 5×10^7^ | 4.33×10^7^ | 1.15×10^7^ | 3×10^8^ | 2×10^8^ | 2×10^8^ | 2.33×10^8^ | 5.77×10^7^ |
| 400 | 5×10^7^ | 4×10^7^ | 5×10^7^ | 4.67×10^7^ | 5.77×10^6^ | 2×10^8^ | 2×10^8^ | 1×10^8^ | 1.67×10^8^ | 5.77×10^7^ |
| 500 | 8×10^6^ | 1×10^7^ | 9×10^6^ | 9.00×10^6^ | 1.00×10^6^ | 8×10^7^ | 8×10^7^ | 1×10^8^ | 8.67×10^7^ | 1.15×10^7^ |
| 750 | 3×10^6^ | 5×10^6^ | 5×10^6^ | 4.33×10^6^ | 1.15×10^6^ | 9×10^7^ | 9×10^7^ | 7×10^7^ | 8.33×10^7^ | 1.15×10^7^ |
| 1000 | 2×10^6^ | 3×10^6^ | 2×10^6^ | 2.33×10^6^ | 5.77×10^5^ | 6×10^7^ | 5×10^7^ | 6×10^7^ | 5.67×10^7^ | 5.77×10^6^ |
| 1500 | 1×10^6^ | 8×10^5^ | 9×10^5^ | 9.00×10^5^ | 1.00×10^5^ | 4×10^7^ | 4×10^7^ | 3×10^7^ | 3.67×10^7^ | 5.77×10^6^ |
| 2000 | 5×10^3^ | 4×10^3^ | 5×10^3^ | 4.67×10^3^ | 5.77×10^2^ | 3×10^5^ | 5×10^5^ | 4×10^5^ | 4.00×10^5^ | 1.00×10^5^ |

M. Splicing-independent resistance (SIR): KD191

| [kan]μg/ml | KD191WT  n=3 | | | KD191WT  mean ±s.d. | | KD191^C118A^  n=3 | | | KD191^C118A^  mean ±s.d. | |
| --- | --- | --- | --- | --- | --- | --- | --- | --- | --- | --- |
| 0 | 5×10^7^ | 4×10^7^ | 5×10^7^ | 4.67×10^7^ | 5.77×10^6^ | 3×10^7^ | 5×10^7^ | 5×10^7^ | 4.33×10^7^ | 1.15×10^7^ |
| 12.5 | 5×10^7^ | 5×10^7^ | 7×10^7^ | 5.67×10^7^ | 1.15×10^7^ | 8×10^6^ | 9×10^6^ | 1×10^7^ | 9.00×10^6^ | 1.00×10^6^ |
| 25 | 5×10^7^ | 6×10^7^ | 7×10^7^ | 6.00×10^7^ | 1.00×10^7^ | 3×10^7^ | 3×10^7^ | 2×10^7^ | 2.67×10^7^ | 5.77×10^6^ |
| 50 | 5×10^7^ | 5×10^7^ | 4×10^7^ | 4.67×10^7^ | 5.77×10^6^ | 3×10^7^ | 3×10^7^ | 4×10^7^ | 3.33×10^7^ | 5.77×10^6^ |
| 75 | 5×10^7^ | 4×10^7^ | 3×10^7^ | 4.00×10^7^ | 1.00×10^7^ | 7×10^6^ | 7×10^6^ | 5×10^6^ | 6.33×10^6^ | 1.15×10^6^ |
| 100 | 4×10^7^ | 3×10^7^ | 4×10^7^ | 3.67×10^7^ | 5.77×10^6^ | 3×10^6^ | 4×10^6^ | 4×10^6^ | 3.67×10^6^ | 5.77×10^5^ |
| 150 | 1×10^7^ | 1×10^7^ | 9×10^6^ | 9.67×10^6^ | 5.77×10^5^ | 5×10^6^ | 5×10^6^ | 2×10^6^ | 4.00×10^6^ | 1.73×10^6^ |
| 200 | 1×10^7^ | 9×10^6^ | 8×10^6^ | 9.00×10^6^ | 1.00×10^6^ | 3×10^6^ | 3×10^6^ | 1×10^6^ | 2.33×10^6^ | 1.15×10^6^ |
| 250 | 1×10^7^ | 1×10^7^ | 9×10^6^ | 9.67×10^6^ | 5.77×10^5^ | 2×10^4^ | 5×10^4^ | 4×10^4^ | 3.67×10^4^ | 1.53×10^4^ |
| 300 | 5×10^6^ | 8×10^6^ | 5×10^6^ | 6.00×10^6^ | 1.73×10^6^ | 6×10^3^ | 3×10^3^ | 2×10^3^ | 3.67×10^3^ | 2.08×10^3^ |
| 400 | 3×10^6^ | 3×10^6^ | 2×10^6^ | 2.67×10^6^ | 5.77×10^5^ | 0 | 0 | 0 | 0 | 0 |
| 500 | 1×10^5^ | 1×10^5^ | 9×10^4^ | 9.67×10^4^ | 5.77×10^3^ | 0 | 0 | 0 | 0 | 0 |
| 750 | 0 | 0 | 0 | 0 | 0 | 0 | 0 | 0 | 0 | 0 |
| 1000 | 0 | 0 | 0 | 0 | 0 | 0 | 0 | 0 | 0 | 0 |
| 1500 | 0 | 0 | 0 | 0 | 0 | 0 | 0 | 0 | 0 | 0 |
| 2000 | 0 | 0 | 0 | 0 | 0 | 0 | 0 | 0 | 0 | 0 |

N. Splicing-independent resistance (SIR): KD242

| [kan]μg/ml | KD242WT  n=3 | | | KD242WT  mean ±s.d. | | KD242^C118A^  n=3 | | | KD242^C118A^  mean ±s.d. | |
| --- | --- | --- | --- | --- | --- | --- | --- | --- | --- | --- |
| 0 | 3×10^7^ | 4×10^7^ | 3×10^7^ | 3.33×10^7^ | 5.77×10^6^ | 1×10^7^ | 1×10^7^ | 2×10^7^ | 1.33×10^7^ | 5.77×10^6^ |
| 12.5 | 1×10^7^ | 1×10^7^ | 9×10^6^ | 9.67×10^6^ | 5.77×10^5^ | 1×10^7^ | 3×10^7^ | 1×10^7^ | 1.67×10^7^ | 1.15×10^7^ |
| 25 | 3×10^7^ | 2×10^7^ | 3×10^7^ | 2.67×10^7^ | 5.77×10^6^ | 8×10^6^ | 9×10^6^ | 1×10^6^ | 9.00×10^6^ | 1.00×10^6^ |
| 50 | 3×10^7^ | 3×10^7^ | 1×10^7^ | 2.33×10^7^ | 1.15×10^7^ | 8×10^6^ | 7×10^6^ | 8×10^6^ | 7.67×10^6^ | 5.77×10^5^ |
| 75 | 1×10^7^ | 9×10^6^ | 1×10^7^ | 9.67×10^6^ | 5.77×10^5^ | 7×10^6^ | 7×10^6^ | 6×10^6^ | 6.67×10^6^ | 5.77×10^5^ |
| 100 | 5×10^6^ | 6×10^6^ | 5×10^6^ | 5.33×10^6^ | 5.77×10^5^ | 5×10^6^ | 5×10^6^ | 4×10^6^ | 4.67×10^6^ | 5.77×10^5^ |
| 150 | 6×10^6^ | 5×10^6^ | 5×10^6^ | 5.33×10^6^ | 5.77×10^5^ | 7×10^6^ | 6×10^6^ | 5×10^6^ | 6.00×10^6^ | 1.00×10^6^ |
| 200 | 3×10^6^ | 4×10^6^ | 2×10^6^ | 3.00×10^6^ | 1.00×10^6^ | 5×10^6^ | 6×10^6^ | 5×10^6^ | 5.33×10^6^ | 5.77×10^5^ |
| 250 | 2×10^6^ | 1×10^6^ | 9×10^5^ | 1.30×10^6^ | 6.08×10^5^ | 3×10^6^ | 3×10^6^ | 3×10^6^ | 3.00×10^6^ | 0 |
| 300 | 1×10^6^ | 9×10^5^ | 1×10^6^ | 9.67×10^5^ | 5.77×10^4^ | 1×10^6^ | 9×10^5^ | 1×10^6^ | 9.67×10^5^ | 5.77×10^4^ |
| 400 | 6×10^5^ | 6×10^5^ | 5×10^5^ | 5.67×10^5^ | 5.77×10^4^ | 1×10^6^ | 2×10^6^ | 9×10^5^ | 1.30×10^6^ | 6.08×10^5^ |
| 500 | 2×10^5^ | 3×10^5^ | 3×10^5^ | 2.67×10^5^ | 5.77×10^4^ | 1×10^5^ | 2×10^5^ | 9×10^4^ | 1.30×10^5^ | 6.08×10^4^ |
| 750 | 0 | 0 | 0 | 0 | 0 | 0 | 0 | 0 | 0 | 0 |
| 1000 | 0 | 0 | 0 | 0 | 0 | 0 | 0 | 0 | 0 | 0 |
| 1500 | 0 | 0 | 0 | 0 | 0 | 0 | 0 | 0 | 0 | 0 |
| 2000 | 0 | 0 | 0 | 0 | 0 | 0 | 0 | 0 | 0 | 0 |

O. No resistance (NR): KD133

| [kan]μg/ml | KD133WT  n=3 | | | KD133WT  mean ±s.d. | | KD133^C118A^  n=3 | | | KD133^C118A^  mean ±s.d. | |
| --- | --- | --- | --- | --- | --- | --- | --- | --- | --- | --- |
| 0 | 7×10^7^ | 6×10^7^ | 7×10^7^ | 6.67×10^7^ | 5.77×10^6^ | 6×10^7^ | 1×10^8^ | 8×10^7^ | 8.00×10^7^ | 1.15×10^7^ |
| 12.5 | 0 | 0 | 0 | 0 | 0 | 0 | 0 | 0 | 0 | 0 |
| 25 | 0 | 0 | 0 | 0 | 0 | 0 | 0 | 0 | 0 | 0 |
| 50 | 0 | 0 | 0 | 0 | 0 | 0 | 0 | 0 | 0 | 0 |
| 75 | 0 | 0 | 0 | 0 | 0 | 0 | 0 | 0 | 0 | 0 |
| 100 | 0 | 0 | 0 | 0 | 0 | 0 | 0 | 0 | 0 | 0 |
| 150 | 0 | 0 | 0 | 0 | 0 | 0 | 0 | 0 | 0 | 0 |
| 200 | 0 | 0 | 0 | 0 | 0 | 0 | 0 | 0 | 0 | 0 |
| 250 | 0 | 0 | 0 | 0 | 0 | 0 | 0 | 0 | 0 | 0 |
| 300 | 0 | 0 | 0 | 0 | 0 | 0 | 0 | 0 | 0 | 0 |
| 400 | 0 | 0 | 0 | 0 | 0 | 0 | 0 | 0 | 0 | 0 |
| 500 | 0 | 0 | 0 | 0 | 0 | 0 | 0 | 0 | 0 | 0 |
| 750 | 0 | 0 | 0 | 0 | 0 | 0 | 0 | 0 | 0 | 0 |
| 1000 | 0 | 0 | 0 | 0 | 0 | 0 | 0 | 0 | 0 | 0 |
| 1500 | 0 | 0 | 0 | 0 | 0 | 0 | 0 | 0 | 0 | 0 |
| 2000 | 0 | 0 | 0 | 0 | 0 | 0 | 0 | 0 | 0 | 0 |

P. No resistance (NR): KD143

| [kan]μg/ml | KD143WT  n=3 | | | KD143WT  mean ±s.d. | | KD143^C118A^  n=3 | | | KD143^C118A^  mean ±s.d. | |
| --- | --- | --- | --- | --- | --- | --- | --- | --- | --- | --- |
| 0 | 2×10^7^ | 5×10^7^ | 4×10^7^ | 3.67×10^7^ | 1.53×10^7^ | 3×10^7^ | 4×10^7^ | 3×10^7^ | 3.33×10^7^ | 5.77×10^6^ |
| 12.5 | 0 | 0 | 0 | 0 | 0 | 0 | 0 | 0 | 0 | 0 |
| 25 | 0 | 0 | 0 | 0 | 0 | 0 | 0 | 0 | 0 | 0 |
| 50 | 0 | 0 | 0 | 0 | 0 | 0 | 0 | 0 | 0 | 0 |
| 75 | 0 | 0 | 0 | 0 | 0 | 0 | 0 | 0 | 0 | 0 |
| 100 | 0 | 0 | 0 | 0 | 0 | 0 | 0 | 0 | 0 | 0 |
| 150 | 0 | 0 | 0 | 0 | 0 | 0 | 0 | 0 | 0 | 0 |
| 200 | 0 | 0 | 0 | 0 | 0 | 0 | 0 | 0 | 0 | 0 |
| 250 | 0 | 0 | 0 | 0 | 0 | 0 | 0 | 0 | 0 | 0 |
| 300 | 0 | 0 | 0 | 0 | 0 | 0 | 0 | 0 | 0 | 0 |
| 400 | 0 | 0 | 0 | 0 | 0 | 0 | 0 | 0 | 0 | 0 |
| 500 | 0 | 0 | 0 | 0 | 0 | 0 | 0 | 0 | 0 | 0 |
| 750 | 0 | 0 | 0 | 0 | 0 | 0 | 0 | 0 | 0 | 0 |
| 1000 | 0 | 0 | 0 | 0 | 0 | 0 | 0 | 0 | 0 | 0 |
| 1500 | 0 | 0 | 0 | 0 | 0 | 0 | 0 | 0 | 0 | 0 |
| 2000 | 0 | 0 | 0 | 0 | 0 | 0 | 0 | 0 | 0 | 0 |

Q. No resistance (NR): KD200

| [kan]μg/ml | KD200WT  n=3 | | | KD200WT  mean ±s.d. | | KD200^C118A^  n=3 | | | KD200^C118A^  mean ±s.d. | |
| --- | --- | --- | --- | --- | --- | --- | --- | --- | --- | --- |
| 0 | 2×10^7^ | 5×10^7^ | 5×10^7^ | 4.00×10^7^ | 1.73×10^7^ | 1×10^8^ | 9×10^7^ | 7×10^7^ | 8.67×10^7^ | 1.53×10^7^ |
| 12.5 | 0 | 0 | 0 | 0 | 0 | 0 | 0 | 0 | 0 | 0 |
| 25 | 0 | 0 | 0 | 0 | 0 | 0 | 0 | 0 | 0 | 0 |
| 50 | 0 | 0 | 0 | 0 | 0 | 0 | 0 | 0 | 0 | 0 |
| 75 | 0 | 0 | 0 | 0 | 0 | 0 | 0 | 0 | 0 | 0 |
| 100 | 0 | 0 | 0 | 0 | 0 | 0 | 0 | 0 | 0 | 0 |
| 150 | 0 | 0 | 0 | 0 | 0 | 0 | 0 | 0 | 0 | 0 |
| 200 | 0 | 0 | 0 | 0 | 0 | 0 | 0 | 0 | 0 | 0 |
| 250 | 0 | 0 | 0 | 0 | 0 | 0 | 0 | 0 | 0 | 0 |
| 300 | 0 | 0 | 0 | 0 | 0 | 0 | 0 | 0 | 0 | 0 |
| 400 | 0 | 0 | 0 | 0 | 0 | 0 | 0 | 0 | 0 | 0 |
| 500 | 0 | 0 | 0 | 0 | 0 | 0 | 0 | 0 | 0 | 0 |
| 750 | 0 | 0 | 0 | 0 | 0 | 0 | 0 | 0 | 0 | 0 |
| 1000 | 0 | 0 | 0 | 0 | 0 | 0 | 0 | 0 | 0 | 0 |
| 1500 | 0 | 0 | 0 | 0 | 0 | 0 | 0 | 0 | 0 | 0 |
| 2000 | 0 | 0 | 0 | 0 | 0 | 0 | 0 | 0 | 0 | 0 |
